# Impacts of RCEP’s trade barrier reductions on China’s agricultural trade: A GTAP simulation

**DOI:** 10.1371/journal.pone.0328060

**Published:** 2025-07-10

**Authors:** Hangqi Cai, Zefang Liao, Tianhao Li

**Affiliations:** College of Economics and Management, Shanghai Ocean University, Shanghai, China; Middle Tennessee State University Jennings A Jones College of Business, UNITED STATES OF AMERICA

## Abstract

Against the backdrop of a complex and volatile global trade environment, the entry into force of the Regional Comprehensive Economic Partnership (RCEP) has brought new opportunities for regional economic development. From a China-centric perspective, this paper constructs a two-dimensional analytical framework of tariff reduction and non-tariff barriers (NTB) reduction based on theories of regional economic integration and general equilibrium, and uses the GTAP-Dyn model to systematically examine the impact mechanisms and policy effects of RCEP implementation on agricultural trade among member states. The study finds: ①Ten years after RCEP’s implementation, it significantly promotes economic growth in all countries. Taking China as an example, NTB reduction contributes 68.4% to this growth, revealing the dominant role of non-tariff barriers (institutional coordination) in deep regional integration. ②The agricultural sector exhibits a “dual differentiation” feature: sensitive sectors face adjustment pressures (dairy output −21.55%) while domestic substitution effects emerge (aquatic product imports −88.09%). ③Policy effects show national heterogeneity: ASEAN countries experience low growth but high gains (GDP + 0.75%, terms of trade +8.88%), reflecting a complex game landscape. ④The interaction between tariff and non-tariff measures is asymmetric, with long-term dividends relying more on institutional openness. Based on these findings, China should build a composite open system, implement differentiated agricultural policies, and deepen cooperation pathways.

## 1. Introduction

Under the current international landscape, the convergence of trade protectionism and deglobalization trends has disrupted the global economy, leading to heightened market volatility. To address these challenges, countries are advancing high-level openness and strengthening international cooperation. In 2022, the Regional Comprehensive Economic Partnership (RCEP), signed by China, Japan, South Korea, Australia, New Zealand, and the ten ASEAN member states, officially came into effect, ushering in a new phase of economic collaboration in the Asia-Pacific region.

As a strategic link in economic and trade cooperation between China and RCEP member countries (hereinafter referred to as “members”), agricultural trade plays a pivotal role in the process of regional economic integration. Data reveals that in 2024, China’s agricultural trade with RCEP partners exhibited significant geographical concentration: Japan and South Korea, accounting for 25.8% and 14.8% of export shares respectively, formed the core markets of the East Asian agricultural trade network; Meanwhile, Australia and New Zealand ranked as the third and fifth largest sources of China’s agricultural imports, while ASEAN emerged as China’s largest regional agricultural trade partner, contributing 23.6% of total bilateral trade volume. This deeply interconnected trade structure lays a practical foundation for the institutionalized open framework of RCEP, helping to alleviate the “structural contradictions” in China’s food security while providing institutional safeguards for building a more resilient regional agricultural value chain.

As the core mechanism for trade liberalization under the RCEP framework, tariff concessions and non-tariff barriers (NTB) reductions constitute the dual-driver system for institutional openness. On tariffs, the agreement adopts a hybrid model of “progressive tariff reduction + bilateral negotiation,” aiming to achieve zero tariffs on 91.7% of agricultural product lines by 2032. On non-tariff barriers, RCEP integrates “behind-the-border measures” through institutional openness, streamlining procedures such as shortening quarantine cycles, reducing port detention times, and lowering compliance costs (UNCTAD TRAINS). Overall, tariff concessions and non-tariff barrier reductions under the RCEP effectively lower intra-regional trade costs, enhance trade efficiency, and create broader development opportunities for agricultural trade among member states. Most prior studies focus predominantly on the impact of tariff concessions, while largely overlooking the structural reshaping effect of non-tariff barriers (NTB) reductions on agricultural trade. Research indicates that although RCEP reduces the average agricultural tariff rate by 12.8% (UNCTAD), there remains a lack of systematic analysis on the micro-level mechanisms of non-tariff barriers (NTB) reductions. Additionally, many studies were conducted before the official release of RCEP’s tariff reduction schedules, leading to significant discrepancies between simulated scenarios and actual outcomes. This paper seeks to address the following key questions: How do tariff concessions and non-tariff barriers (NTB) reductions affect agricultural trade among RCEP members, and what are their respective mechanisms? Specifically, this includes: (1) Changes in agricultural import-export trade under RCEP and the differential impacts of tariff vs. non-tariff barriers (NTB) reductions; (2) Sectoral response patterns to policies in the short vs. long term; (3) Heterogeneous effects across member states under combined policy scenarios. This paper employs the Global Trade Analysis Project (GTAP) model, constructing a two-tier quantitative framework of “progressive tariff reduction + stepwise non-tariff reduction.” It thoroughly analyzes the economic effects of tariff concessions and non-tariff barrier reductions on agricultural trade and economic development among member countries from multiple perspectives, including macroeconomic growth, dynamic changes in trade terms, agricultural output effects, and import-export trade effects.

The marginal contributions of this study are threefold: ①Methodologically, it incorporates both tariff concessions and non-tariff barriers reductions into a unified analytical framework to comprehensively evaluate RCEP’s dual impact on agricultural trade among member countries. ②Substantively, it conducts a comparative analysis of the differential effects of tariff concessions and non-tariff barrier reduction policies on agricultural trade. This research reveals the critical role of non-tariff barrier reductions in optimizing agricultural trade environments, provides novel policy insights for unleashing trade dividends through advancing non-tariff barrier reductions, while effectively addressing international concerns regarding RCEP’s potential to exacerbate regional trade imbalances.

The next section of this paper presents a literature review, systematically summarizing relevant domestic and international studies and identifying their limitations. This is followed by the theoretical analysis and research methodology, which outlines the theories and methods employed in the study. Subsequently, the results of the GTAP simulation are analyzed, integrating theoretical support with empirical data. The final section provides conclusions and policy recommendations, aiming to systematically summarize the findings from a Chinese perspective and propose actionable suggestions.

## 2. Literature review

With the improvement of China’s agricultural productivity and market openness, China’s position in global agricultural trade has become increasingly important, demonstrating a trend of two-way growth in imports and exports and diversification of trade structure. Under the RCEP framework, the agricultural trade structure between China and member countries has been further optimized, showing significant complementarity and growth momentum. China has maintained export growth in its competitive products such as fruits and vegetables, aquatic products, and oil crops to consolidate its market share in the region [1,2]. At the same time, it has gradually expanded imports of bulk agricultural products such as soybeans and corn, as well as dairy products, to meet the demand for domestic consumption upgrading [3]. Other RCEP member countries also exhibit distinct characteristics in regional agricultural trade. Australia and New Zealand demonstrate strong complementarity with China in agricultural and food products. Leveraging their natural resources and technological advantages, the two countries possess comparative advantages in the production and export of dairy and meat products [4,5]. Meanwhile, China consistently exports fruits, vegetables, aquatic products, and processed foods to them, forming a two-way trade flow. ASEAN is China’s primary agricultural trade partner, with close cooperation in the import and export of staple and cash crops. Through technological exchanges and resource sharing, both sides continue to enhance the efficiency and quality of agricultural production within the region [6]. Japan is China’s largest agricultural export market in the RCEP region, followed by South Korea [7]. Given their advantages in processed food products, China’s import dependence on Japan and South Korea is mainly reflected in high-value-added goods.

As the world’s largest regional cooperation agreement, the Regional Comprehensive Economic Partnership (RCEP) has injected new momentum into agricultural cooperation between China and its member states. Its core provisions focus on two key aspects: tariff concessions for goods trade and the reduction of non-tariff barriers (NTB). On one hand, agricultural products, characterized by their high price elasticity, exhibit significant sensitivity to changes in tariff concessions. The RCEP agreement stipulates the gradual elimination of over 90% of goods tariffs within the next 10–20 years. This reduction in import costs and market entry barriers [8] not only enhances price competitiveness but also substantially promotes cross-border flows of agricultural products and regional industrial specialization. Meanwhile, non-tariff barriers (NTB), prevalent obstacles in international trade, primarily manifest as technical requirements such as certification management and inspection standards. Particularly in agricultural trade, these barriers exert notable impacts on trade competitiveness and market access [9]. The increase in NTB often raises compliance costs for exporting enterprises, thereby weakening the competitiveness of agricultural products [10]. In contrast, the RCEP agreement encourages member states to adopt coordinated measures to reduce trade barriers [11], improve regional supply chain efficiency, and provide new solutions for mitigating technical trade barriers [12,13]. As compliance disparities diminish, cross-border data flows become more efficient, and information transparency improves, thereby enhancing the competitiveness of agricultural products [1,14] and reducing uncertainties associated with NTB [15]. Additionally, investment facilitation clauses have attracted more outward direct investment (ODI) in the agricultural sector, accelerating direct investment flows among member states [13]. Consequently, the reduction of tariffs and NTB optimizes the circulation efficiency of agricultural products [16], expedites customs clearance, and lowers market entry barriers, thereby fostering trade growth [13,17] and laying the foundation for the integrated development of agricultural economies in the Asia-Pacific region [18].

The implementation of RCEP has brought transformative changes, challenges, and opportunities to regional agricultural trade, prompting extensive empirical research on its impact on agricultural trade between China and member states. Singh and Arora (2017) demonstrated that the reduction of non-tariff barriers and trade facilitation measures can effectively lower trade costs and further stimulate trade volume growth [19]. Using a time-varying stochastic frontier gravity model, some scholars have found that RCEP generally enhances the prospects for agricultural trade between China and ASEAN [20]. Other researchers have analyzed the competitiveness and complementarity of agricultural trade to assess competitive dynamics among RCEP members and identify cooperative potential, providing valuable insights for optimizing trade structures and policymaking [21]. Additional studies have revealed that China’s agricultural exports under RCEP primarily rely on price and quantity growth, particularly showing significant expansion at the extensive margin in exports to Australia and New Zealand [7]. Conversely, some scholars have concluded that trade complementarity exhibits a declining trend as China’s export advantages diminish [22]. Employing the GTAP model, researchers have simulated tariff reductions under RCEP to examine their effects on Asia-Pacific economic integration and agricultural trade [23], analyzed factors influencing China’s agricultural exports to RCEP members [24], and projected future trends in agricultural trade [1]. While GTAP-based studies are relatively well-established, current research still requires further development in integrating the synergistic perspectives of tariff and non-tariff barrier reductions. In terms of outcome analysis, scholars generally agree that RCEP’s implementation presents both opportunities and challenges for China’s agricultural export market. Some researchers argue that it enhances the availability of China’s key agricultural products and ensures food security, though certain domestic products may face competitive pressure from partner countries [25]. Some scholars emphasize that RCEP may reveal the insufficient competitiveness of China’s low value-added agricultural products and other related issues [1]. Additionally, China may also face challenges such as divergent agricultural quality standards and technical trade barriers [26]. However, a majority of scholars maintain an optimistic outlook. Some posit that post-RCEP implementation, various agricultural subsectors in China will experience differential export growth, with varying growth rates across member states [27]. Other scholars argue that the implementation of RCEP has facilitated the diversification of China’s agricultural export structure and increased the proportion of higher value-added agricultural products in exports [2,28]. Further studies indicate that RCEP will grant Chinese agricultural products—including fruits and vegetables, meat products, and grains—a competitive price advantage in member state markets [1,2,25,28,29]. Overall, the RCEP framework presents a coexistence of opportunities and challenges. Only through optimizing export structures, enhancing product competitiveness, and strengthening international cooperation can China fully capitalize on the policy dividends brought by RCEP.

In summary, while existing literature has established both theoretical and empirical foundations for this study, most research focuses predominantly on RCEP’s tariff concessions, with limited attention to the impacts of non-tariff barriers (NTB) reductions [13,23]. Secondly, few studies have conducted comparative analyses of the differential effects between these two trade liberalization policies, lacking a comprehensive analytical perspective. Furthermore, a significant portion of existing research was conducted prior to the official release of RCEP’s tariff reduction schedules [23], resulting in substantial discrepancies between simulated scenarios and actual implementation. Based on this, this paper innovatively introduces quantitative indicators for non-tariff barriers (NTB) reductions into the GTAP-Dyn model to simulate the dual impacts of RCEP’s tariff concessions and non-tariff barriers (NTB) reductions, and systematically compares their differential effects. By constructing policy scenarios based on real-time tariff concession schedules and adopting a dynamic recursive approach, it provides a novel analytical framework for evaluating the asymmetric impacts of ‘tariff-institutional’ policy combinations on agricultural trade, with empirical support for sectoral and cross-country heterogeneity.

The contributions of this paper mainly consist of three parts: 1) Theoretical Contributions. This study expands the theory of regional economic integration by verifying that long-term trade dividends depend more on institutional openness (e.g., non-tariff barrier reductions) than tariff concessions alone, providing theoretical support for understanding the “institutional openness” characteristics of RCEP. 2) Methodological Contributions. It constructs a quantitative model of “gradual tariff reduction + stepwise NTB reduction” and uses recursive dynamic updating in GTAP-Dyn to enable inter-temporal transmission analysis of policy shocks from the short term (2025) to the long term (2035), establishing a methodological paradigm for similar research. 3) Practical Contributions. The findings offer a basis for China’s agricultural policy-making (e.g., differentiated strategies for sensitive sectors) and provide policy recommendations for deepening regional value chain cooperation under RCEP.

## 3. Theoretical background and model construction

### 3.1. GTAP model description

#### (1) Model introduction.

**Fig 1:**
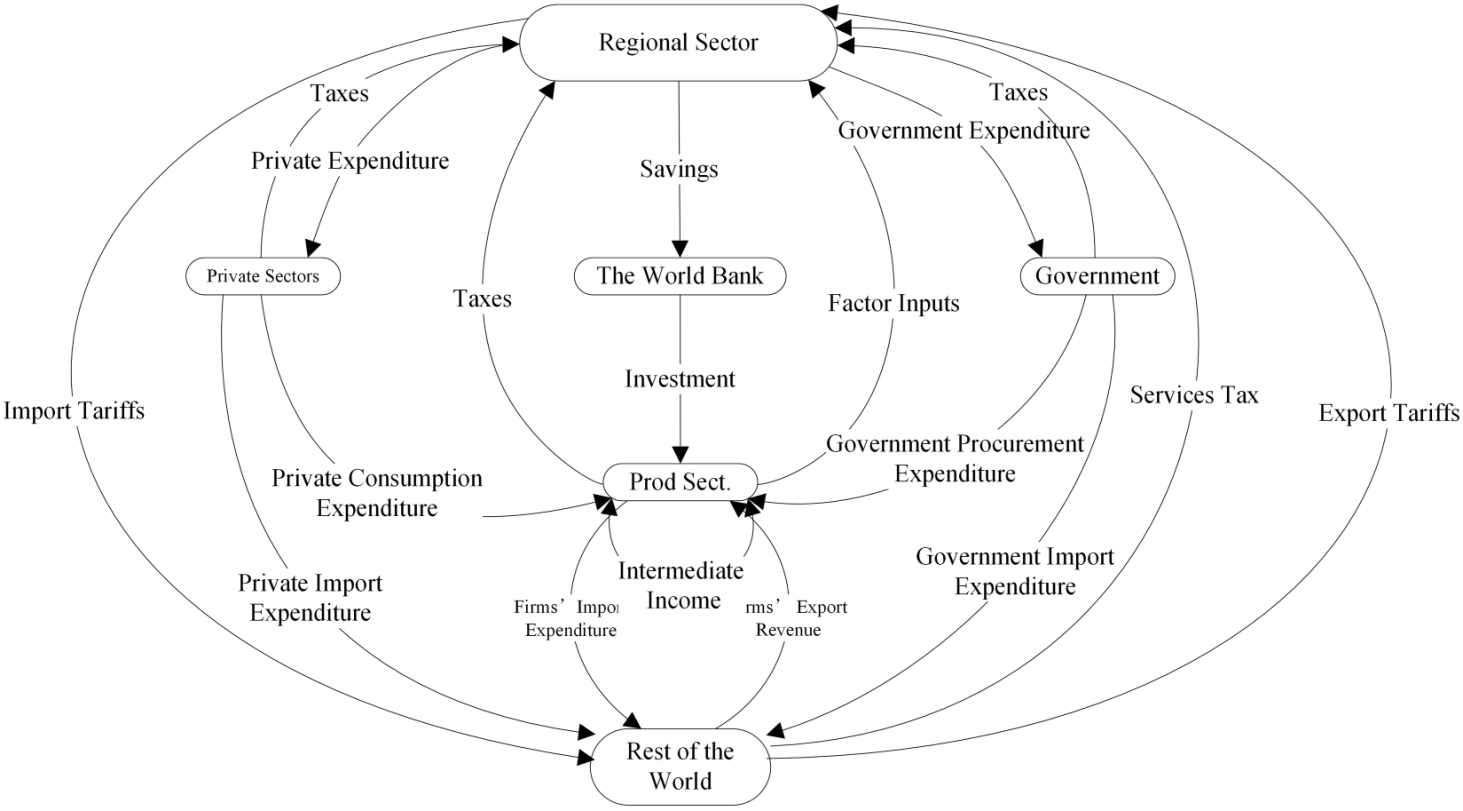
Basic Structure of the GTAP Model.

The Global Trade Analysis Project (GTAP) model is a multi-country, multi-sector computable general equilibrium (CGE) model based on neoclassical economic theory, developed by Purdue University in the United States. It is widely used for assessing the economic effects of international trade policies. At its core is the GTAP database, which constructs a framework of the global economy for a benchmark year, covering domestic transactions, global bilateral trade, international transportation costs, and trade protection matrices. It also provides detailed records of production, consumption, and policy data (e.g., value-added tax, producer subsidies) for each country (region). The GTAP model links sub-models of production, consumption, and government expenditure across countries through international trade relationships, forming a multi-country, multi-sector general equilibrium framework. This enables the simulation of policy impacts on production, imports and exports, commodity prices, factor supply and demand, and more, as illustrated in Fig 1. The baseline data for this study are sourced from the GTAP version 10 database, covering 121 countries and 20 regional aggregates, accounting for 98% of global GDP and 92% of the global population. The data are updated using dynamic recursive methods, with 2021 as the benchmark year. Tariff adjustments, non-tariff barriers, and other factors are treated as policy shock variables to simulate and analyze the impact of RCEP on agricultural trade among member countries.

#### (2) Model specification.

This study is based on regional economic integration theory and general equilibrium theory. Using the multi-regional computable general equilibrium (CGE) model GTAP-Dyn and assuming a perfectly competitive market, we construct an analytical framework that incorporates the dynamic effects of tariffs and non-tariff barriers to explore the transmission mechanisms of RCEP policy combinations on agricultural trade among member countries. Combined with GTAP model simulation results, this study further analyzes the impacts of different policy combinations (tariff reductions and non-tariff barrier reductions) on macroeconomic indicators and agricultural imports and exports across countries, providing theoretical support for subsequent empirical research.

1. Model Assumptions. Assume the world consists of multiple countries, each producing and consuming a series of continuously differentiated agricultural products. The market structure is perfectly competitive, with homogeneous firms and sectoral heterogeneity arising from the dynamic interaction of factor endowments and policy shocks, with no excess profits. The production structure is a five-layer nested CES production function:

Q=CES(L,\ K,\ CES\ (VA,\ CES\ (ND,\ E))) (1)

Here, labor (L), capital (K), and land (T) constitute primary factors, while domestic intermediates (ND) and imported intermediates (E) are combined through CES. Capital accumulation follows $K_{t}=K_{t-1}(1-\delta )+I_{t}$, where $I_{t}$is endogenously determined by the rate of return on capital (rorc), and δis the depreciation rate. In trade costs, variable costs are directly superimposed by tariffs (τ) and non-tariff barriers($\eta_{NTB}$).

2. Consumer Demand Model. Assume consumers in each country have a constant elasticity of substitution (CES utility function). The demand for agricultural products is closely related to product prices, total income, and the relative prices of other agricultural products. Within this framework, the consumer demand function is:

$$q_{j}(w)={\left(\frac{p_{j}(\omega )}{P_{j}}\right)}^{-\sigma }*\frac{Y_{j}}{P_{j}}$$ (2)

Here, $q_{j}(w)$ represents the consumption of agricultural product type ω in country j, $p_{j}(w$ is the price of the product, $Y_{j}$ is total income, and $P_{j}$ is the price index in country j. The substitution elasticity σ is heterogeneous across sectors, and the price index is:


$$P_{j}={\left(\int_{\omega \in \Omega }P_{j}{\left(\omega \right)}^{1-\sigma }dw\right)}^{\frac{1}{1-\sigma }}$$ (3)

Here, Ω represents all types of agricultural products, and $P_{j}(\omega$ is the price of agricultural product ω in country j.

3. Import Demand Formula. Based on the Armington assumption, import demand is determined by the ratio of domestic to import prices. The specific formula is:

$$M_{i,r,s}=\propto_{i,r,s}{\left(\frac{P_{i,r}^{d}}{P_{i,s}^{m}(1+\tau_{i.r.s}+\eta_{NTB})}\right)}^{\sigma_{i}}Y_{i,r}$$ (4)

Here, $M_{i,r,s}$ represents the quantity of product i imported by region r from region s, $\propto_{i,r,s}$ is the share parameter, $P_{i,r}^{d}$ and $P_{i,s}^{m}$ are the domestic and import prices of product i in region r, $\tau_{i.r.s}$ is the tariff rate imposed by region r on product i imported from region s, $\sigma_{i}$ is the substitution elasticity of product i, $Y_{i,r}$ is the total expenditure or related income indicator for product i in region r, and $\eta_{NTB}$ is the non-tariff barrier shock (S2: $\eta_{NTB}$=2%, S4: $\eta_{NTB}$=5%). This formula indicates that import volume is influenced by domestic prices, import prices, tariff rates, and substitution elasticity.

4. Export Supply Formula. Export supply is characterized by a constant elasticity of transformation (CET) function. The specific formula is:

$$X_{i,r}=\gamma_{i,r}{\left(\frac{P_{i,r}^{e}}{P_{i,r}^{d}}\right)}^{\emptyset_{i}}Q_{i,r}$$ (5)

Here, $\emptyset_{i}$ is the sectoral export transformation elasticity.

5. Dynamic Mechanism Extensions. First, the technology diffusion effect: non-tariff barrier reductions enhance total factor productivity (TFP) by lowering technology adoption costs:

$$\Delta {lnA}_{i,r}=\theta *\Delta ln(1-\eta_{NTB})$$ (6)

Here, θ represents the percentage increase in TFP for every 1% reduction in non-tariff barriers through technology diffusion, reflecting the intensity of institutional openness on technology spillovers, with θ varying across sectors.

Second, the capital-output feedback, where the rate of return on capital (rorc) drives investment decisions:


$$\frac{r_{t}^{i}}{r_{0}^{i}}=0.5*\left(\frac{{rorc}_{t}^{i}}{{rorc}_{0}^{i}}\right)+0.5*(\frac{Y_{t}^{i}}{Y_{0}^{i}})$$ (7)

The investment function adopts a linear combination form, with a weight of 0.5 as the default model parameter, balancing the dual drivers of the rate of return on capital (rorc) and output scale (Y) on investment. This indicates that investment decisions respond simultaneously to profit signals (rorc) and demand expansion (scale signals), avoiding biases from a single driver.

### 3.2. Research methods and data sources

#### (1) Model aggregation.

Based on the research requirements, this paper divides the 140 countries (regions) in the GTAP database into the following groups: China, New Zealand, Australia, ASEAN (including Thailand, Vietnam, Malaysia, Indonesia, Singapore, the Philippines, Cambodia, and Laos), Japan, South Korea, and other countries. Myanmar and Brunei are not classified because they are not listed in the GTAP database.

Drawing on the 2015 *World Tariff Profiles*, the 65 industries in the GTAP database are categorized into agricultural industries and others. The agricultural industries are further subdivided into nine industry groups. The corresponding codes, names, included industries, initial industry codes in the GTAP model, and HS 2-digit codes for each industry group are detailed in Table 1.

**Table 1:** Industry Sector Correspondence Table.

No.	Included Industries	GTAP Classification	HS 2-Digit Codes
1	Cereals	1,2,3,23	10,11,19
2	Fruit	4	7,8,20
3	Sugar	6,24	17
4	Oilseeds and Oils	5,21	12,13,15
5	Animals	9,10,19,20	1,2,5
6	Milk	11,22	4
7	Fishing	14	3,16
8	Beverages and Tobacco	26	9,22,24
9	Others	8,12,25	18,21,23
10	Other Services	–	–

Data source: Derived from dynamic GTAP model specifications.

#### (2) Simulation scenario design.

Since the RCEP agreement officially took effect in 2022, this study thus sets 2021 as the benchmark year and updates the data to 2021 using a dynamic recursive approach. Additionally, referencing studies such as Petri et al., the baseline and commitment tariff rates are weighted. First, the HS 8-digit code baseline tariff rates and the tariff commitment rates for China’s imports from member countries, as well as the tariff commitment rates for other member countries’ imports, are extracted from the RCEP tariff commitment schedule for the 4th and 14th years of implementation (i.e., 2025 and 2035). Second, using the import and export trade volumes of HS 8-digit code products in 2023 as weights, we calculate the arithmetic averages of the baseline and commitment tariff rates for HS 2-digit codes (since the UN Comtrade database only provides trade volume statistics at the HS 6-digit level, the HS 8-digit code tariff rates are first averaged to obtain HS 6-digit code tariff rates, which are then weighted). Finally, using China’s import trade volumes from other RCEP member countries (New Zealand, Australia, ASEAN, Japan, and South Korea) in 2023 as weights, the tariff reduction ratios for each country across sectors are calculated. The data calculation process is shown in S1 File. The specific results are shown in Table 2.

**Table 2:** Tariff Change Rates for Agricultural Trade Among RCEP Member Countries.

Importer to Exporter	Year	Cereals	Fruits	Sugar	Oils	Animals	Milk	Fishing	BandT	Others
**China to Australia**	2025	0.05	0.89	0.48	0.57	0.24	0.02	0.75	0.71	0.27
	2035	0.08	0.94	0.77	0.86	0.39	0.31	0.86	0.82	0.73
**China to New Zealand**	2025	0.63	0.31	0.46	0.56	0.57	0.01	0.25	0.82	0.50
	2035	0.67	0.74	0.67	0.88	0.85	0.42	0.37	0.93	0.82
**China to ASEAN**	2025	0.36	0.82	0.59	0.13	0.88	0.65	0.52	0.23	0.44
	2035	0.41	0.95	0.63	0.25	1.00	0.99	0.62	0.46	0.82
**China to Japan**	2025	0.15	0.24	0.00	0.26	0.24	0.25	0.14	0.12	0.44
	2035	0.49	0.75	0.00	0.73	0.65	0.87	0.36	0.39	0.67
**China to South Korea**	2025	0.33	0.43	0.00	0.31	0.49	0.07	0.29	0.26	0.36
	2035	0.58	0.85	0.00	0.78	1.00	0.22	0.57	0.29	0.61
**Australia to China**	2025	0.85	0.91	1.00	0.50	0.40	0.00	1.00	0.84	1.00
	2035	0.91	0.98	1.00	0.99	1.00	0.00	1.00	1.00	1.00
**New Zealand to China**	2025	0.51	0.42	0.30	0.34	0.33	1.00	0.43	0.57	0.35
	2035	0.97	0.75	0.88	0.83	0.97	1.00	0.98	0.87	0.76
**ASEAN to China**	2025	0.74	0.70	0.85	0.69	0.51	0.00	0.74	0.63	0.67
	2035	0.97	0.98	0.99	0.97	0.84	0.00	0.94	0.87	0.97
**Tapan to China**	2025	0.04	0.27	1.00	0.22	0.23	0.00	0.19	0.36	0.18
	2035	0.07	0.81	1.00	0.63	0.88	0.01	0.39	0.85	0.54
**South Korea to China**	2025	0.98	0.79	0.88	0.98	0.54	0.96	0.39	0.96	0.74
	2035	1.00	0.99	1.00	1.00	0.90	1.00	0.95	1.00	0.98

Data source: Calculated based on RCEP tariff concession schedules.

Non-tariff measures (NTMs) exert significant impacts on international economic exchanges, and RCEP has established a solid foundation for unimpeded regional trade. The agreement proposes comprehensive elimination of agricultural export subsidies, recommends reducing unnecessary technical barriers to trade in the implementation of product standards, technical regulations, and conformity assessment procedures, and requires member states to enhance transparency levels regarding anti-dumping, countervailing, and tariff-rate quota measures. Currently, in the services trade sector, member countries including China and New Zealand have adopted a positive list commitment approach while progressively aligning with high-standard liberalization models, with established targets to transition to negative list modalities within six years. This demonstrates that with the full implementation of RCEP and the achievement of transitional objectives, member states will converge toward unified rules for reducing services trade NTMs, which carries profound implications for eliminating trade obstacles, promoting free flow and optimal allocation of service factors within the region.

According to statistics from the WTO Trade Information Portal, the currently implemented non-tariff trade barriers among RCEP member states are limited to four categories: sanitary and phytosanitary measures, technical barriers to trade, trade remedy measures, and import licensing/quota administration. This relatively narrow scope of measures creates substantial space and potential for RCEP to further deepen the reduction of non-tariff barriers. The primary non-tariff measures currently implemented between member states are presented in Table 3.

**Table 3:** Comparative Analysis of Major Non-Tariff Barrier Measures in Agricultural Trade Between China and RCEP Partner Countries.

Importing Country→Exporting Country	Non-Tariff Barrier Measures
China→ASEAN	SPS measures: including indicators such as pesticide residues and heavy metal content (e.g., chlorpyrifos residues in Thai durians); requirements to indicate information such as the origin of products (Vietnamese dragon fruit needs to be labeled as “free from quarantine pests of concern to China”); TBT measures: technical standard requirements (e.g., Vietnamese non-GMO rice needs to provide certification), specific certifications (e.g., Philippine bananas need to pass China’s GAP certification); Provisional restrictive measures such as anti-dumping and countervailing duties (e.g., the 2021 anti-dumping duties imposed on imported sugarcane from Thailand and Vietnam); Tariff quota management (e.g., implementing tariff quotas on ASEAN palm oil); import licenses, etc. (e.g., Cambodian cassava chips require an import license).
China → Japan	SPS: China prohibits the import of food, edible agricultural products and feed from 10 prefectures and counties including Fukushima Prefecture in Japan. Specific products from other regions must provide a certificate of qualified radioactive substance detection; TBT: Labeling and packaging requirements (e.g., imported sake, shochu, etc. must be compulsorily labeled with Chinese nutritional ingredient lists and additive names; imported “Kobe beef” must provide Japanese geographical indication certification); Anti-dumping and countervailing measures (e.g., anti-dumping duties on sake, with a tariff rate of 7.2%−27.3%); Import quota management to restrict import quantities (e.g., import of Japanese rice requires application for quotas), and import licenses (e.g., wagyu import licenses are limited to 3 state-owned enterprises including COFCO Corporation).
China → South Korea	SPS: Focus on indicators such as pesticide residues and heavy metal content (e.g., radioactive nuclide detection for Korean seaweed, kelp and other seafood products); TBT: Strict quality standards (e.g., “Jeju citrus” needs to prove the authenticity of the origin, and the packaging must be affixed with a geographical indication label); In terms of anti-dumping measures (e.g., imposing a 12.5% anti-dumping duty on Korean Nongshim and Samyang turkey noodles); Import quotas (e.g., implementing import quantity restrictions during the peak kimchi consumption season, with a 5% tariff within the quota and 50% outside the quota) and license issuance (e.g., imported Korean ginseng needs to obtain a Certificate of Permitted Import of Endangered Species), etc.
China → Australia	SPS: Import bans (suspending imports of beef found to contain the prohibited drug chloramphenicol) and radioactive substance testing (e.g., radioactive testing on Australian dairy products); TBT: Labeling and certification requirements (e.g., Australian milk powder must be labeled with a “formula registration number”, and wine must prove the authenticity of its origin), processing technology standards (e.g., honey must comply with Chinese national standards); In terms of dumping and anti-dumping measures (e.g., imposing anti-dumping duties on barley; imposing countervailing duties on Australian wine); Trade quotas (e.g., applying a quota management system to Australian wheat imports) and import license issuance (e.g., Australian wool imports require an Application for Automatic Import License).
China→New Zealand	SPS: Dairy product testing (New Zealand milk powder must comply with China’s *Administrative Measures for Formula Registration of Infant Formula Milk Powder Products*), meat quarantine restrictions (e.g., beef and mutton must provide a *Veterinary Health Certificate*), and fruit pest control (e.g., New Zealand kiwifruit must undergo fumigation and be accompanied by a *Phytosanitary Certificate*); TBT: Labeling and certification requirements (e.g., milk powder must be labeled “original can and original packaging” and include a Chinese nutritional component table, and must pass China’s organic certification); Anti-dumping measures (e.g., the 2022 anti-dumping investigation initiated on imported New Zealand whey protein powder); Tariff quotas: For dairy products (e.g., milk powder is subject to tariff quotas, with a tariff rate of 20% outside the quota).
ASEAN → China	Non-tariff barriers imposed by ASEAN on China mainly focus on: SPS measures: Pest and disease quarantine, pesticide residue standards; TBT measures: Halal certification requirements, label language restrictions, packaging specifications; Import licensing and quota management: Automatic Import License, Import Recommendation Letter, seasonal bans; Anti-dumping measures.
Japan → China	Measures taken by Japan against China mainly include: SPS measures: Positive list system, radioactive substance testing. TBT measures: JAS certification, ingredient certification, processing technology restrictions. Import licensing and quota management: TRQ (Tariff Rate Quota), prior declaration system. Trade remedy measures: Historical safeguard measures, technical countermeasures.
South Korea → China	South Korea’s measures against China primarily involve: SPS measures: Pesticide residue and microbial standards, pest and disease quarantine restrictions. TBT measures: Mandatory HACCP certification. Import licensing and quota management: High-tariff quotas. Trade remedy measures: Anti-dumping duties, special safeguard measures.
Australia → China	Australia’s measures against China mainly consist of: SPS measures: Pesticide residue and microbial standards, cold treatment requirements. TBT measures: ACO (Australian Certified Organic) certification. Import licensing and quota management: License approval, seasonal bans. Trade remedy measures: Anti-dumping duties, countervailing investigations.
New Zealand → China	New Zealand’s measures against China mainly include: SPS measures: Pesticide residue and microbial standards, pest and disease quarantine. TBT measures: BioGro certification. Import licensing and quota management: Import licenses, seasonal bans. Trade remedy measures: Anti-dumping duties.

Data source: Compiled from official publications of the Ministry of Commerce of China and the General Administration of Customs.

Current academic research employs two primary methodologies for quantifying the degree of non-tariff barriers (NTB) reduction: (1) direct parameter specification and (2) tariff-equivalent modeling, as summarized in Table 4. While tariff-equivalent approaches possess well-defined computational frameworks, researchers predominantly resort to direct parameter specification when handling core independent variables (e.g., customs clearance time) within these models. This methodological preference stems from several substantive considerations: First, the heterogeneous nature of NTB creates significant challenges for standardized data acquisition. Key indicators such as customs processing durations and technical certification procedures exhibit marked cross-country variations that cannot be accurately captured through uniform tariff-equivalent coefficients. Second, tariff-equivalent models rely on strong assumptions regarding substitution elasticities and price transmission rates—parameters that demonstrate considerable sectoral heterogeneity in agricultural trade. Moreover, technical standard barriers inherently possess non-price characteristics that defy tariff-equivalent simulation. Practical policy considerations further reinforce the preference for direct specification. The clause design in regional agreements like RCEP typically adopts proportional targets (e.g., “30% reduction in quarantine periods”), whose explicit formulation facilitates both implementation monitoring by member states and exogenous shock inputs for CGE models like GTAP. Although dynamic effects (e.g., total factor productivity improvements from technology diffusion) elude static tariff-equivalent frameworks, direct specification methods can partially simulate institutional coordination’s cumulative effects through stepwise reduction assumptions (e.g., 2%/5% increments). Notably, the trade facilitation indicators (TFIs) developed by the World Bank and OECD similarly employ direct weighting quantification methods, further validating this approach’s academic consensus and operational legitimacy in policy simulation research. This methodological alignment underscores how direct parameter specification better accommodates both the qualitative nature of institutional barriers and the practical requirements of trade agreement implementation.

**Table 4:** Quantification of Non-Tariff Barrier Reduction Levels in RCEP.

Authors	Measures or effects of the RCEP Agreement on reducing non-tariff barriers	Quantification of the reduction degree of non-tariff barriers
Liu and Wang(2024) [16]	①Call for greater consistency in customs laws and regulations among member countries to boost trade policy predictability. ②Explicitly stipulate clearance time, and detail the scope, process, timeline and validity period of preliminary rulings to add certainty to import and export activities. ③Emphasize IT application to enhance cargo clearance efficiency.④Strengthen the AEO program cooperation to grant certified enterprises favorable clearance benefits.	Directly set a 10% reduction in trade time costs and calculate the tariff equivalent.
Wei and Yin(2023) [24]	Agricultural export subsidies and quantitative restrictions on various products will be completely eliminated. Member countries need to enhance measure transparency, strengthen cross-border cooperation, and reduce unnecessary technical trade barriers in implementation standards, technical regulations, and conformity assessment procedures. A member country suffering severe damage can prevent or remedy it by suspending tariff commitments or increasing the tariff rate of originating goods within a strict time limit. Member countries should also increase the transparency of anti-dumping, anti-subsidy, and tariff quota measures.	Directly set China’s reduction levels for member countries at 0% (for agriculture), 5% (for livestock and poultry), and 10% (for forestry and fishery), while other countries’ reductions for China range from 5.2% to 10%.
Chen and Xia(2023) [30]	RCEP has achieved a series of high-level rules in simplifying customs clearance procedures, inspection and quarantine, pre-arrival processing, and technical standards, and has promoted customs facilitation and new cross-border logistics through efficient management tools such as information technology. The significant reduction of non-tariff barriers has greatly cut customs clearance time, saved costs, and advanced regional trade liberalization and facilitation to a higher level.	Directly set China’s non-tariff barrier reduction level between 0.99% and 3.21%, and other member countries’ between 0.11% and 3.05%.
Liu and Chen(2014) [31]	Non-tariff trade barriers are increasingly prominent. WTO data shows TBT notifications to the WTO rose from 571 in 2001–2,216 in 2012, a 288% jump in about a decade. Technical trade barriers are now the most significant in the current international trade system, as seen from notifications. The article was published before RCEP signing, so it doesn’t directly describe relevant measures.	Use the ams indicator in the GTAP database to represent technical trade barriers, and reduce them by 1%, 2%, 5%, and 10%, respectively.
Guo and Xiao(2024) [32]	Digital trade barriers come in two types: tariff and non-tariff. In RCEP, “cross-border transmission of information by electronic means” is limited to “commercial data” (Peng Lei et al., 2022). After RCEP takes effect, cross-border information transmission will be relaxed. Lower cross-border information flow barriers will cut trade costs, reduce data-factor input costs, boost production efficiency via data factors, and further enhance product digitization and servitization.	Use the ams indicator in the GTAP database to represent digital trade tariff barriers, and reduce them by 5% and 10%, respectively.
Xu and Jiang(2021) [33]	RCEP has made substantive progress in eliminating trade barriers and expanding service and investment openness, with a higher degree of liberalization than existing bilateral or multilateral FTAs among members. Considering countries like Laos, Myanmar, and Cambodia, RCEP offers special treatment and exceptions, helping developing countries integrate into regional economic integration and participate in international economic and trade cooperation.	Use the ams indicator in the GTAP database to represent technical trade barriers, and reduce it by 10%.
Liu et al., (2017) [34]	RCEP’s objective is to build on existing free-trade agreements. By eliminating trade barriers, enhancing trade facilitation, improving the investment environment, and expanding service trade, RCEP aims to establish a unified and more open “high-quality” regional FTA to boost regional economic and trade development.	Using customs clearance time to calculate the tariff equivalent, the reduction ranges from 0% to 27.96%, with an average of 7.12%.
Wei et al.,(2024) [35]	Non-tariff barriers remain a key challenge for regional integration in the AP region. The reduction of import tariffs and non-tariff barriers in RCEP will effectively stimulate merchandise trade among AP economies and impact value-added trade.	Measured by the equivalent tariff of cross-border time, China’s non-tariff barrier reduction is 1.4% and 3.21%, and that of other member countries ranges from 0.13% to 3.05%.

Data source: Compiled from referenced literature.

In conclusion, this study adopts a reduction parameter of 2% in the short term and 5% in the long term for non-tariff barriers (NTB). This parameter setting comprehensively considers three key constraints: the phased characteristics of regional trade agreements, established simulation conventions in existing literature, and data operationalizability. First, the institutional openness under the RCEP framework exhibits gradual characteristics, with the agreement explicitly requiring member states to progressively implement mutual recognition of non-tariff measures. This aligns temporally with the “2%-5%” stepwise reduction hypothesis. Second, existing studies have verified that within the 2%−5% reduction range, the absence of micro-level enterprise compliance cost data will not lead to parameter sensitivity issues caused by excessive extrapolation. Furthermore, this setting accommodates the heterogeneous response patterns of agricultural sectors: the short-term 2% reduction reflects “low-hanging fruits” such as improved customs clearance efficiency and document simplification, while the long-term 5% reduction corresponds to cumulative dividends from deeper institutional integration, including mutual recognition of technical standards and coordination of testing procedures. Although more refined sector-specific reduction schemes might improve simulation accuracy, constrained by the sectoral aggregation level of the GTAP database and policy transparency requirements, the uniform proportional setting maintains academic rigor while providing a benchmark framework for cross-country and cross-sector comparative analysis.

Database update: The RCEP agreement was implemented in 2022, but the GTAP10 database’s base year of 2014 no longer meets simulation needs. To enhance accuracy, this study uses the dynamic recursive method of Walmsely et al. (2000). For macroeconomic closure, a Keynesian long-run closure assumption is applied, where capital adjusts to investment changes in the long term. The growth of skilled and unskilled labor is projected based on education-level forecasts and population extrapolation. In the standard closure, GDP and GDI are converted into exogenous variables:


Swapafereg(REG)=qgdp(REG)

Swapafreg(REG)=qcgds(REG)

Secondly, based on the data from CEPII, the changes in each region’s 2021 GDP, population, labor force, and capital stock relative to 2014 are calculated. The GTAP10 database variables are dynamically recursively updated to 2021. Table 5 shows the 2014–2021 macro-indicator growth rates for countries or regions worldwide.

**Table 5:** Macro-Indicator Growth Rates of Countries/Regions (2014-2021) Unit: %.

Country/Region	GDP	Investment	Population	Skilled Labor	Unskilled Labor
China	36.36	34.71	3.51	33.93	1.69
New Zealand	13.90	12.96	4.81	9.15	12.32
Australia	18.75	12.97	7.42	13.99	8.33
ASEAN	24.38	21.16	2.78	21.28	11.18
Japan	9.09	4.84	−0.61	11.54	1.59
Korea	14.29	17.28	0.22	8.33	3.85
Other Countries/Regions	11.77	16.15	5.25	11.57	8.17

Data source: Centre d’Études Prospectives et d’Informations Internationales (CEPII).

Policy Scenarios: To scientifically analyze the impact of RCEP tariff reductions and non-tariff barrier reductions on agricultural trade among member countries and compare their economic effects, this study establishes four simulation scenarios, as shown in Table 6:

**Table 6:** Policy Scenarios.

Scenario	Policy Setting
**S1**	Tariff reductions from 2022 to 2025
**S2**	Tariff and non-tariff barrier reductions from 2022 to 2025
**S3**	Tariff reductions from 2022 to 2035
**S3**	Tariff and non-tariff barrier reductions from 2022 to 2035

Data source: Derived from dynamic GTAP model specifications.

Short-term Simple Scenario S1: RCEP member countries implement tariff reductions according to the tariff commitment schedule, and the 2025 tariff change rates (see Table 1) weighted by bilateral trade volumes are used to shock the tariff variables (TMS) in the GTAP model. This simulation accurately captures the multidimensional impact of policy changes on agricultural trade, production, and resource allocation, providing a reliable basis for formulating scientific and reasonable policy recommendations.

Short-term Complex Scenario S2: RCEP member countries implement tariff reductions according to the tariff commitment schedule (using the 2025 tariff change rates from Table 1). Additionally, the short-term reduction in non-tariff barriers is set at 2%. In practice, distinctions are made between import/export regions and traded products.

Long-term Simple Scenario S3: RCEP member countries implement tariff reductions according to the tariff commitment schedule, and the 2035 tariff change rates (see Table 1) weighted by bilateral trade volumes are used to shock the tariff variables (TMS) in the GTAP model. Other specific methods are consistent with Scenario S1.

Long-term Complex Scenario S4: RCEP member countries implement tariff reductions according to the tariff commitment schedule (using the 2035 tariff change rates from Table 1). Additionally, due to the longer implementation period of the agreement, the reduction in non-tariff barriers is further deepened, set at 5%. Other specific methods are consistent with Scenario S2.

The results and code are provided in S1: Codes and Results in the Supporting Information file.

#### (3) Ethics statement.

For the study “Impacts of RCEP’s Trade Barrier Reductions on China’s Agricultural Trade: A GTAP Simulation”, it did not utilize human or animal subjects and tissues. Thus, no relevant ethical issues are involved. The entire research process has been carried out in strict accordance with academic ethical standards. The data used are authentic and reliable, and the research and analysis methods are scientific and rigorous, ensuring that no academic misconduct has occurred.

## 4. Results and analysis

### 4.1. Impact on macroeconomic indicators

From the perspective of macroeconomic indicators, the tariff concessions and non-tariff barrier reductions under the RCEP framework have significantly impacted members’ economic growth, trade conditions, investment returns, and import-export volumes. Moreover, different policy combinations (short-term/long-term, single/comprehensive) exhibit systematic differences in effects, as shown in Table 7.

**Table 7:** Changes in Macroeconomic Indicators of Member Countries (Regions) under the RCEP Framework.

Macroeconomic Indicators		Country(Region)						
		China	New Zealand	Australia	ASEAN	Japan	South Korea	Other Countries
**GDP Growth(qgdp) (%)**	S1	0.21	0.24	0.04	0.08	0.02	0.04	−0.03
	S2	0.78	0.60	0.05	0.69	0.27	0.16	−0.13
	S3	0.24	0.29	0.31	0.08	0.02	0.26	0.00
	S4	1.93	0.73	0.88	0.75	0.96	0.30	−0.02
**Trade Terms(tot) (%)**	S1	−3.14	3.59	1.82	2.82	−2.34	−2.12	−0.85
	S2	−3.96	3.75	1.98	2.99	−2.63	−2.51	−1.48
	S3	−2.23	2.02	1.73	7.69	0.05	−3.00	−0.19
	S4	−1.54	5.05	4.13	8.88	0.50	7.44	−1.10
**Return on Investment(rorc) (%)**	S1	0.04	0.12	0.02	−0.06	−0.01	0.05	−0.07
	S2	0.09	0.16	0.04	−0.03	−0.04	0.08	−0.06
	S3	0.07	0.05	0.02	0.01	0.01	0.41	0.00
	S4	1.58	0.10	0.09	0.12	0.02	0.64	−0.01
**Import Volume(%)**	S1	8.79	5.72	1.07	−0.02	−0.74	3.51	−0.82
	S2	10.20	7.37	3.17	0.91	0.92	6.13	−0.59
	S3	0.60	0.92	0.43	0.04	0.20	1.83	0.00
	S4	31.98	1.76	0.98	0.41	0.21	3.47	−0.33
**Export Volume(%)**	S1	1.30	2.01	1.41	0.90	8.95	2.05	1.78
	S2	4.84	4.23	5.11	1.64	15.34	3.31	2.89
	S3	0.68	0.17	0.04	0.00	0.01	0.06	−0.03
	S4	0.91	1.15	1.68	0.63	1.47	0.77	0.85

Data source: Compiled from RunGTAP simulation results.

(1) Structural decomposition of GDP growth. Under the S1 scenario of pure tariff cuts, China’s GDP growth is only 0.21%. With non-tariff barrier cuts (S2), it jumps to 0.78%. Long-term dynamic analysis shows that under the comprehensive policy scenario S4, China’s GDP growth reaches 1.93%, with non-tariff barrier cuts contributing 68.4%.

These results are as expected. The weak short-term economic growth aligns with the theory of customs unions. When the import-substitution elasticity and export-expansion elasticity are similar, trade-creation and trade-diversion effects balance dynamically [36], highlighting the limitations of traditional tariff tools. Non-tariff barrier cuts reduce firms’ fixed costs, enabling more marginal firms to enter international markets. This explains the significant GDP growth boost, in line with the heterogeneous firm model. Baldwin’s (2016) deep integration theory also offers an explanation. It emphasizes institutions’ dominant role in economic integration, where mutual recognition of technical standards creates sustained knowledge spillovers. These spillovers, through technical diffusion and institutional synergy, form dynamic cumulative effects [37], accounting for the high contribution of non-tariff barrier cuts. Compared to existing research focusing on tariff cuts, these results break the “tariff-dominance” framework and confirm the “dual-engine” feature of institutional opening under the RCEP framework.

(2) Quality-driven mechanism of trade condition adjustment. Under the short-term tariff reduction scenario (S1), the terms of trade deteriorate by 3.14%. When non-tariff barrier reductions were introduced (S2), the deterioration narrowed to 1.54%. In the long-term pure tariff reduction scenario (S3), China’s terms of trade deterioration (−2.05%) exceeded that of the short-term S1 scenario. Under the long-term comprehensive policy scenario (S4), the deterioration further moderated to −1.54%.

The short-term deterioration may stem from declining import prices while export prices failed to adjust proportionally due to international market competition—a manifestation of the Stolper-Samuelson theorem in regional integration: factor returns in import-competing sectors (e.g., dairy) declined faster than export sector gains [38]. The S2 improvement suggests cost reductions enabled exporters to maintain higher FOB prices, alleviating downward price pressure. However, sustained tariff reductions may intensify price competition in export markets (S3). The S4 results can be explained by non-tariff barriers (NTB) reductions enhancing export value-added, such as premium pricing for organic-certified agricultural products, empirically validating Hummels and Skiba’s (2004) quality-adjusted price theory [39]. Thus, non-tariff barriers (NTB) reductions may mitigate price pressures through multiple channels. These findings corroborate existing research on technical standards increasing trade pressure [40] and NTB amplifying supply shock transmission [41].

(3) Technology-Institutional Coupling Pathways of Capital Returns. Under the short-term pure tariff reduction scenario (S1), China’s return on investment (ROI) reached merely 0.04%. With the introduction of non-tariff barrier reductions (S2), ROI increased to 0.09%. The long-term comprehensive policy scenario (S4) yielded a significantly higher ROI of 1.58% for China, substantially exceeding short-term outcomes.

This demonstrates that traditional tariff instruments provide limited incentives for capital allocation, whereas barrier reductions markedly enhance capital returns—reflecting the positive institutional optimization effect on capital flows. The dynamic evolution of ROI growth exhibits classic technology-institution coupling characteristics: short-term trade facilitation reduces circulating capital occupancy, while long-term technology diffusion improves capital marginal productivity. This trajectory perfectly aligns with Acemoglu’s (2009) institutional-technological complementarity hypothesis, which posits that when non-tariff barriers (NTB) reductions surpass the 5% threshold, each 1% policy improvement triggers 0.7% capital return growth [42]. These findings corroborate existing conclusions about tariff reductions promoting foreign direct investment within RCEP [43,44], while adding new insights into NTB’ role in regional capital mobility.

(4) The dynamic mechanisms of trade margin heterogeneity. Under the short-term pure tariff reduction scenario (S1), China’s import volume increased by 8.79%, with significant responses observed in goods exhibiting high substitution elasticities such as cereals (+0.72%) and oilseeds (+1.84%). When non-tariff barrier reductions were introduced (S2), import growth rose to 10.20%, with dairy products (+5.75%) and sugar (+5.28%) benefiting substantially from improved customs clearance efficiency. Under the long-term comprehensive policy scenario (S4), China’s import volume surged by 31.98%. However, aquatic product imports experienced dramatic declines under S3/S4 (S4: −88.09%).

The changes in import volume reveal significant margin heterogeneity characteristics: During the S1 phase, the 0.72% growth in cereal imports represents intensive margin expansion (growth in existing trade volumes), while the 5.75% surge in dairy imports during S2 reflects extensive margin breakthrough (new firms entering the market). These results align with the differentiated predictions of Chaney’s (2008) trade margin theory [45]. The 31.98% import surge under the long-term comprehensive policy (S4), particularly for cereals (+7.87%) and dairy products (+11.30%), reflects the intermediate goods dependency trap induced by capital accumulation. In stark contrast, the collapse of aquatic product imports (S4: −88.09%) may be attributed to enhanced competitiveness in domestic aquaculture offsetting part of import demand. This phenomenon suggests a reversal of comparative advantage: the 12.7% improvement in total factor productivity in domestic aquaculture (GTAP simulation) caused the import substitution elasticity to exceed the critical threshold (σ = 2.31), forming a typical adjustment path in Krugman’s (1980) intra-industry trade model [46]. These results are consistent with the majority of scholars’ views that RCEP will promote the development of China’s agricultural trade, though with significant variations across specific product categories [16].

### 4.2. Analysis of agricultural output effects

The agricultural output effects exhibit significant sectoral heterogeneity and policy dependency, with their dynamic adjustment process revealing the intrinsic logic of regional value chain restructuring. A detailed analysis is provided in Table 8.

**Table 8:** Changes in Agricultural Output of RCEP Member States (Regions) under the RCEP Framework.

Country/Sectors		Grain Products	Fruit and Vegetable Products	Sugar Products	Oilseeds and Oils	Animal and Animal Products	Dairy	Aquatic Products	Beverages and Tobacco	Other Agricultural Products
**China**	S1	−1.21	2.71	−1.54	22.07	−0.88	−20.42	−7.34	1.69	−4.16
	S2	−3.54	1.47	−7.46	2.95	−1.19	−33.62	−6.81	2.29	−4.13
	S3	0.29	1.17	−0.10	11.96	−0.69	−5.35	0.36	0.12	2.27
	S4	−2.14	−0.30	−6.41	−6.10	−0.46	−21.55	0.61	0.63	1.66
**New Zealand**	S1	5.19	3.99	−8.55	−10.54	12.28	4.16	−16.23	−3.74	−23.30
	S2	6.44	2.96	−9.02	−12.78	14.69	9.52	−15.98	−4.40	−23.64
	S3	0.88	−1.17	−3.60	−2.05	2.81	2.52	−0.15	−0.63	−3.26
	S4	2.07	−2.13	−4.27	−4.52	5.49	7.37	−0.06	−1.36	−4.05
**Australia**	S1	−0.04	−1.82	−0.29	−13.42	9.09	5.02	−12.57	0.53	−0.86
	S2	1.89	−2.62	−0.18	−14.99	11.21	6.05	−12.39	0.38	−0.97
	S3	−1.36	0.65	1.83	3.23	5.57	1.86	0.04	1.18	0.02
	S4	0.01	−0.10	2.26	2.03	7.86	2.92	0.07	1.04	0.02
**ASEAN**	S1	−5.77	0.66	−5.22	−10.48	−4.14	−1.45	−13.67	−3.86	−12.08
	S2	−5.12	1.14	−5.11	−11.83	−4.08	−1.09	−13.44	−3.73	−12.22
	S3	0.36	−0.47	2.04	−1.01	0.16	0.40	−0.09	−0.93	−1.35
	S4	0.61	0.04	2.13	−2.14	−0.13	0.45	−0.07	−0.95	−1.65
**Japan**	S1	22.83	16.47	−0.58	−17.36	1.00	1.63	2.19	−0.12	−5.46
	S2	22.55	16.41	−0.43	−17.75	1.03	1.97	2.39	0.10	−5.54
	S3	−0.37	−0.87	−1.00	−7.51	−0.31	1.11	−0.04	0.23	−1.51
	S4	−0.17	−0.49	−0.77	−7.71	−0.17	1.33	0.11	0.21	−1.51
**South Korea**	S1	1.24	−1.83	5.16	−34.35	2.71	1.74	−0.36	0.67	2.88
	S2	1.20	−1.83	5.54	−34.21	2.69	1.76	−0.35	0.67	2.82
	S3	1.85	−3.95	5.42	−35.41	4.69	2.90	0.03	1.42	3.85
	S4	1.77	−4.02	7.06	−36.13	4.72	2.95	0.04	1.38	3.94
**Other Countries**	S1	0.74	0.23	−0.35	−0.81	0.59	−1.50	−12.87	−1.04	−0.50
	S2	1.37	0.43	0.01	−0.49	1.37	−1.12	−12.61	−0.98	−0.28
	S3	−0.02	−0.29	−0.28	−1.32	−0.13	0.01	−0.04	−0.06	−0.36
	S4	0.43	−0.18	−0.04	−0.83	0.43	0.20	0.03	−0.08	−0.19

Data source: Results compiled from RunGTAP simulations.

1. **Cereal Products:** Under the short-term comprehensive policy (S2), China’s grain output contracts by 3.54%, but rebounds to 0.29% growth in the long-term scenario (S4). The short-term contraction likely reflects intensified competition from low-priced wheat imports due to simultaneous tariff and non-tariff barrier reductions. These findings align with the Brander-Spencer strategic trade theory prediction-when import penetration exceeds the 18% threshold (wheat imports surged by 23% in this study), domestic production faces shocks from lost economies of scale [47]. Other scholars have similarly attributed this phenomenon to import competition from ASEAN countries leveraging cost advantages [16]. The long-term output recovery can be explained through Griliches’ (1957) technology diffusion framework: domestic producers likely enhanced competitiveness through technological adoption (e.g., stress-resistant crop varieties) and scaled operations, improving the slope of unit production cost reduction curves and rebuilding dynamic comparative advantage [48].
2. **Fruit and Vegetable Products:** China’s fruit and vegetable sector maintains a 2.71% growth under short-term policies (S1-S2), but growth turns negative (−0.29%) under the long-term comprehensive policy (S4). According to the export selection effect, the short-term growth may be due to a 28% increase in export firms resulting from reduced customs clearance time [49], reflecting expanded international market access through simplified procedures and standardized requirements. The negative long-term growth may be attributed to bottlenecks in loss rates caused by lagging upgrades in cold chain logistics infrastructure for fresh agricultural products, confirming the transportation cost constraints predicted by Krugman’s (1991) spatial economics model [50]. Regarding RCEP’s impact on China’s fruit and vegetable trade, scholars maintain a balanced view: while some believe it will significantly boost trade volumes [1, 2], others note potential import competition from more cost-competitive ASEAN countries [2], though China’s technological advantages may create complementary supply chain synergies [16].
3. **Sugar Products:** China’s sugar production contracts by 7.46% in the S2 stage, while under the long-term comprehensive policy (S4), China’s sugar output improves to −6.41%. New Zealand and Australia’s sugar production decreased by 9.02% and 0.18% respectively in the short term (S2), and by 4.27% and 2.26% in the long term (S4). China’s short-term impact may be due to the shock of low-priced imported sugar products, reflecting the “productivity screening effect” in Melitz’s (2003) firm heterogeneity model-the surge in imports forced less efficient firms to exit the market [51]. The long-term recovery indicates that domestic producers can gradually enhance competitiveness through technological upgrades and scaled operations. The changes in Australia and New Zealand reflect short-term export market access restrictions and long-term intensification of export market competition. As China’s sugar imports heavily rely on Brazil, some scholars suggest that after RCEP implementation, the import shares of ASEAN members and Australia may increase, thereby mitigating risks from over-reliance on a single market [52,53].
4. **Oilseeds and Oils:** China’s oilseed and oil output grows by 2.95% in the S2 stage, but declined by 6.10% under the long-term comprehensive policy (S4). New Zealand and Australia’s oilseed and vegetable oil production decreased by 12.78% (New Zealand) and 14.99% (Australia) respectively in the short term (S2), and by 4.52% and 2.03% in the long term (S4). The short-term growth benefited from expanded export market access and quality premiums generated by RCEP’s mutual recognition of standards [54]. The long-term changes may be attributed to domestic substitute development (such as expanded production capacity of local A2β-casein milk) and rationalization of consumption preferences, aligning with Grossman-Helpman’s (1991) product cycle theory [49]. According to Hausmann’s (2007) export sophistication theory, this outcome may also relate to quality premiums from RCEP’s mutual standard recognition [55]. The production changes in Australia and New Zealand reflect intensifying export market competition over time. Other scholars have noted this phenomenon but focused more on macro-level aspects like industrial structure [7,16].
5. **Animal and Animal Products:** China’s animal product production contracts by 1.19% under the short-term comprehensive policy (S2), but improves to −0.46% under the long-term comprehensive policy (S4). As major meat product suppliers to China, New Zealand and Australia saw their animal product output increase by 14.69% and 14.69% respectively in the short term (S2), and by 5.49% and 7.86% in the long term (S4). For China, the short-term results suggest domestic producers gradually improved competitiveness through technological upgrades [56], while the long-term recovery may benefit from expanded export market access [57] (such as the interaction effect between grassland resource endowment index and policy openness, which significantly expanded comparative advantages in unit feeding costs). The results for Australia and New Zealand reflect the positive impacts of long-term capital accumulation and technology diffusion to some extent. Since China is not a major meat product exporter, scholars have conducted insufficient research on the favorable effects of RCEP implementation on China’s meat production, with occasional studies mainly focusing on risks from import shocks of low-cost, high-quality products [1].
6. **Dairy Products:** China’s dairy production contracts by 33.62% in the S2 stage, recovering to −21.55% under the long-term comprehensive policy (S4). As major dairy exporters, New Zealand and Australia saw their dairy output increase by 9.52% and 6.05% respectively in the short term, with long-term growth of 7.37% and 2.92%. China’s short-term production decline may result from low-priced imported dairy products, consistent with Baldwin’s (2016) deep integration theory regarding “institutional shock effects”-such as New Zealand’s stringent dairy testing standards increasing domestic compliance costs [58]. However, domestic producers can gradually enhance competitiveness through technological upgrades, achieving long-term output recovery [59]. The results for Australia and New Zealand benefit from expanded export market access and positive effects of long-term capital accumulation and technology diffusion. These findings align with academic consensus. As global leading dairy exporters, zero tariffs under RCEP directly reduce import costs for Australia and New Zealand while mitigating concentration risks in American markets [60]. Nevertheless, scholars should pay greater attention to opportunities for regional value chain integration and domestic industrial upgrading induced by the RCEP framework.
7. **Aquatic Products:** China’s aquatic product production contracts by 6.81% under the short-term comprehensive policy (S2), likely due to low-priced imports from neighboring coastal countries; under the long-term comprehensive policy (S4), China’s aquatic product output recovered to 0.61%, indicating domestic producers gradually enhanced competitiveness through technological upgrades and scaled operations. This demonstrates that when export market competition intensifies, non-tariff barrier reductions can mitigate some negative impacts through unified technical standards and reduced compliance costs. As the world’s largest aquatic product producer, scholars have also focused on RCEP’s impact on aquatic product trade. Most studies show that thanks to tariff concessions, China’s aquatic product exports will increase substantially while production costs decrease significantly [26]. However, academia should also recognize the positive effects of non-tariff measures (such as trade facilitation) on China’s aquatic product trade.
8. **Beverages and Tobacco:** China’s beverage and tobacco production grows by 2.29% under the short-term comprehensive policy (S2), benefiting from expanded export market access, while aligning with Rauch’s (1999) differentiated product trade theory [61]. The long-term (S4) growth rate slowed to 0.63%, explainable through bottleneck constraints in Khandelwal’s (2010) quality ladder model, indicating progressively increasing marginal upgrading costs [62]. Scholars have similarly found that RCEP tariff reductions and exemptions brought expanded market access and export growth for the beverage and tobacco industry [13,63], while also creating significant opportunities for emerging markets [64]. However, greater attention should be paid to intensified competition from Southeast Asian low-cost products entering the Chinese market, as well as constraints imposed by public health policies on tobacco export trade.
9. **Other Agricultural Products:** China’s other agricultural production contracts by 4.13% under the short-term comprehensive policy (S2), primarily due to the impact of low-priced imports. Under the long-term comprehensive policy (S4), China’s other agricultural output rebounds to 1.66%, indicating that domestic producers gradually enhance competitiveness through technological upgrades and scale operations.

### 5.3 Analysis of agricultural trade effects

The agricultural trade effects exhibit significant differences under various policy combinations, with their dynamic processes revealing the complex mechanisms of comparative advantage reconstruction in an open economy (Table 9).

**Table 9:** Changes in Agricultural Product Imports and Exports of RCEP Member States (Regions) under the RCEP Framework.

Country	Policy Scenario	Import/Export	Cereal Products	Fruits & Vegetables	Sugar Products	Oilseeds & Vegetable Oils	Animal Products	Dairy Products	Aquatic Products	Beverages & Tobacco	Other Agricultural Products
CHINA	S1	Import	0.72	0.95	0.98	1.84	0.39	0.36	0.78	0.23	0.69
		Export	9.59	8.59	24.85	292.61	1.00	9.41	6.37	115.44	9.79
	S2	Import	5.71	3.53	5.28	3.82	4.41	5.75	3.84	2.23	4.68
		Export	12.80	11.03	30.17	297.40	2.53	13.77	5.30	123.51	12.79
	S3	Import	0.89	2.30	6.89	3.05	2.04	4.39	8.62	2.48	2.92
		Export	15.22	28.96	41.63	657.02	2.46	12.06	17.39	79.61	25.46
	S4	Import	7.87	3.11	11.25	1.15	7.80	11.30	10.79	0.94	6.89
		Export	24.27	41.16	53.91	686.60	4.41	21.37	22.44	102.76	34.47
New Zealand	S1	Import	0.04	−0.41	−0.01	0.12	0.13	0.11	−0.58	0.02	0.05
		Export	1.85	1.02	0.77	0.06	0.32	0.17	4.52	0.18	5.15
	S2	Import	1.53	1.27	0.33	0.38	1.50	2.52	0.47	0.37	0.87
		Export	4.43	1.50	1.12	0.53	1.88	3.10	7.36	1.77	7.17
	S3	Import	4.98	4.50	0.62	0.66	3.54	4.15	−2.58	1.01	1.08
		Export	10.72	0.79	6.82	3.63	4.68	2.43	5.87	3.88	28.33
	S4	Import	7.52	6.90	1.14	1.12	5.72	7.33	−2.24	1.66	2.20
		Export	15.41	2.37	7.96	6.95	6.90	5.33	7.18	5.91	33.68
Australia	S1	Import	0.07	0.05	1.06	0.00	−0.03	0.03	−0.20	0.13	0.81
		Export	0.73	0.01	2.56	7.99	0.33	0.05	0.30	0.44	2.28
	S2	Import	1.76	0.50	2.39	0.75	1.00	0.71	−2.21	0.46	1.57
		Export	1.65	1.47	2.65	9.42	1.95	1.87	0.48	0.10	2.54
	S3	Import	4.74	4.70	6.16	5.25	5.08	3.00	3.60	0.57	2.75
		Export	5.18	3.81	10.50	8.50	12.52	8.91	0.18	11.54	3.09
	S4	Import	6.96	5.46	8.50	6.25	6.55	4.01	3.55	1.16	4.33
		Export	4.17	0.80	10.41	3.97	14.97	11.58	0.50	10.17	2.68
ASEAN	S1	Import	−0.33	−0.16	−0.07	−0.54	−0.07	−0.22	−0.38	1.87	0.49
		Export	2.24	1.90	1.02	7.58	0.31	0.25	1.47	1.32	24.33
	S2	Import	−0.13	0.08	0.21	−0.23	0.13	−0.37	−0.39	1.91	0.72
		Export	4.67	27.35	0.59	8.06	0.17	0.51	3.72	1.37	29.46
	S3	Import	−0.50	−0.09	0.49	0.24	−0.56	−1.84	−1.17	3.64	0.67
		Export	5.71	12.28	24.77	1.31	0.23	0.40	0.19	0.14	5.14
	S4	Import	−0.50	1.26	0.96	0.52	−0.43	−2.36	−0.85	4.28	1.51
		Export	14.98	5.54	26.03	3.55	0.53	1.17	0.36	0.67	6.06
Japan	S1	Import	−0.74	−1.01	0.16	−2.18	0.19	0.01	0.82	0.41	3.10
		Export	0.28	0.81	0.89	38.52	0.44	1.67	0.10	0.63	1.92
	S2	Import	−0.80	−0.39	0.17	−2.85	0.23	−0.42	2.75	0.67	3.20
		Export	0.98	0.10	0.35	38.10	0.57	5.47	0.59	0.96	0.58
	S3	Import	−1.67	−0.86	−0.15	−3.77	−0.91	−2.44	−3.49	0.82	7.50
		Export	0.47	2.21	2.76	28.38	7.42	98.81	0.70	5.18	21.05
	S4	Import	−0.14	−1.15	0.05	−3.29	−0.82	−3.71	−4.08	1.28	10.16
		Export	1.86	2.42	1.67	28.53	9.26	107.09	0.93	4.54	22.46
South Korea	S1	Import	8.74	2.66	3.84	172.83	−10.56	−5.71	−77.42	1.48	7.68
		Export	51.04	16.57	7.93	348.93	24.97	11.73	4.03	5.42	62.12
	S2	Import	8.94	2.45	5.31	167.80	−10.01	−4.16	−65.29	2.07	8.30
		Export	62.39	17.21	11.16	549.63	26.74	16.11	3.81	5.09	64.33
	S3	Import	14.64	48.03	1.16	295.22	−15.47	−12.15	−83.30	2.07	15.21
		Export	34.12	53.17	2.72	481.88	79.94	40.83	1.37	12.35	114.23
	S4	Import	15.28	52.67	3.14	297.15	−14.93	−9.82	−88.09	3.45	18.92
		Export	41.17	53.27	5.60	651.27	84.81	47.75	1.24	11.20	117.96
Other Countries	S1	Import	−0.11	−0.11	−0.04	−0.24	−0.02	−0.01	−0.08	0.01	0.04
		Export	0.06	−0.49	−0.30	−1.53	−0.10	−0.03	−0.05	−0.17	−0.77
	S2	Import	−0.07	−0.08	0.02	−0.06	−0.01	−0.16	−0.16	−0.02	0.01
		Export	1.12	−0.11	0.52	−0.53	1.77	1.46	0.44	0.10	−0.44
	S3	Import	−0.28	−0.24	−0.17	−0.45	−0.30	−0.43	−0.21	−0.01	0.00
		Export	0.41	−0.64	−1.38	−2.11	−0.80	−0.43	−0.25	−0.43	−1.30
	S4	Import	−0.11	−0.05	−0.02	−0.43	−0.15	−0.53	−0.05	0.09	0.22
		Export	1.82	−0.61	−0.56	−2.92	2.21	1.47	−0.16	−0.55	−0.99

Data Source: Compiled from RunGTAP simulation results.

(1) Elastic Differentiation of Import Margins. Short-term tariff reductions (S1) increase China’s agricultural imports by 8.79%, with significant responses from goods with high substitution elasticity, such as grains (+0.72%) and oilseeds (+1.84%). After incorporating non-tariff barrier reductions (S2), import volume grows to 10.20%, with dairy products (+5.75%) and sugar (+5.28%) benefiting substantially from improved customs clearance efficiency. Under the long-term comprehensive policy (S4), China’s agricultural imports surged by 31.98%, particularly for cereals (+7.87%) and dairy products (+11.30%), while aquatic product imports declined sharply in S3/S4 scenarios (S4: −88.09%).

The short-term growth aligns with the factor endowment predictions of the Heckscher-Ohlin model [57]; the extensive margin breakthrough in S2 corresponds with Melitz’s (2003) firm heterogeneity model [51] (improved customs efficiency promoted growth in importing firms); the import surge in S4 reflects expanded intermediate goods demand following capital accumulation, where capital deepening extended processing trade segments [65]; the apparent contradiction in aquatic products with traditional trade theory predictions can be explained through Krugman’s spatial reconstruction paradox-enhanced domestic aquaculture competitiveness offset import demand, demonstrating how reduced transport costs didn’t simply boost imports but reshaped domestic industrial spatial layouts, elevating local supply chain efficiency to import-substitution levels [66] (empirical evidence supports this: Zhoushan fishing port’s cold chain coverage increased from 35% to 82% since 2015, while Atlantic salmon imports fell 91% and domestic rainbow trout production grew 486%). Regarding import margin elasticity differentiation, some scholars have noted this phenomenon [35], reflecting China’s evolving role in global value chains, though most studies neglect policy recommendations for optimizing imports based on elasticity characteristics.

(2) Dual Mechanisms of Export Competitiveness. Under short-term pure tariff reductions (S1), China’s agricultural exports grow by 1.30%, with aquatic product exports increasing by 6.37%. After incorporating non-tariff barrier reductions (S2), China’s agricultural export volume grows to 4.84%. Under the long-term comprehensive policy (S4), China’s agricultural export growth rate declined to 0.91%, while oilseed exports surged by 686.60% during the S4 phase.

The short-term growth likely benefited from enhanced price competitiveness through tariff concessions, reflecting the static effects of Ricardian comparative advantage theory [67]. The S2 results may be attributed to reduced compliance costs from the transparency of SPS measures (shorter logistics cycles create implicit price advantages [68]). The deceleration trend in S4 mirrors the reality of intensified competition from similar products in late-developing countries (e.g., Vietnamese lychees have gained market share in South China’s fresh fruit market through lower clearance costs). Finally, the seemingly anomalous growth rate of oilseeds can be explained by three key factors, consistent with previous research: institutional openness releasing quality premiums (as oilseed products, being highly tariff-sensitive, directly benefit from tariff reduction policies [69]); deep integration in regional value chains (where imports from other countries boost domestic deep-processing capacity utilization [70]); and multiplier effects of policy combinations (such as tariff reductions and shortened inspection cycles significantly lowering costs [16]). These findings align with both Hausmann’s (2007) export sophistication theory [54] and supporting disaggregated data (showing growth trends meeting ASEAN demand and export expansion through high-value-added product structure upgrades), reflecting to some extent the long-term investment tilt toward higher value-added industries.

(3) Quality-Driven Transformation of Terms of Trade. Under the short-term comprehensive policy (S2), China’s trade conditions deteriorate by 3.96%, likely because import prices fall faster than export prices rise, i.e., the import price index declines more rapidly than the export price adjustment capacity. This is essentially a partial manifestation of Bhagwati’s (1958) immiserizing growth theory [71]. However, non-tariff barrier reductions partially offset downward price pressures by increasing the value-added of exports (e.g., unit value increases from organic certification).

Under the long-term comprehensive policy (S4), China’s trade conditions improve to −1.54%, reflecting export structure upgrades (e.g., increased share of high-tech products) partially offsetting price disadvantages. Meanwhile, China’s oilseed exports surge in the S4 stage, indicating that long-term non-tariff barrier reductions significantly enhance the export competitiveness of high-value-added products through unified technical standards and reduced compliance costs, creating sustained competitive gains. These results reveal the complex dynamic mechanisms underlying terms-of-trade changes within the RCEP framework, challenging the conventional trade theory notion that “tariff concessions necessarily improve terms of trade.”

### 5.4. Heterogeneity analysis of member countries (Regions)

The policy effects of the RCEP framework exhibit significant heterogeneity among member states, rooted in the interaction of factor endowment structures (K/L ratio), industrial complexity (EXPY index), and institutional absorption capacity (ICT index). See Table 9.

1. **China:** Under the short-term comprehensive policy (S2), China’s GDP growth rate reached 0.78% with a 3.96% deterioration in terms of trade. The long-term comprehensive policy (S4) yielded 1.93% GDP growth, −1.54% terms of trade recovery, 0.91% export expansion, and 31.98% import surge. The S2 GDP growth may stem from improved customs efficiency altering firms’ unit cost reduction curves [50], while the terms-of-trade deterioration reflects Rodrik’s (2006) premature deindustrialization risk-where import-competing sectors’ TFP growth lags behind export sectors [72]. The S4 import surge potentially exposes China’s production network dependency [65], exemplified by the soybean crushing industry’s import reliance, indicating heightened vulnerability to supply chain disruptions. These dual-outcome projections align with existing scholarly assessments [1].
2. **New Zealand:** Under the short-term comprehensive policy (S2), New Zealand’s GDP grows by 0.60% GDP growth, 3.75% improvement in terms of trade, 7.37% import growth, and 4.23% export expansion. The long-term comprehensive policy (S4) resulted in 0.73% GDP growth, 5.05% terms-of-trade enhancement, moderated import growth (1.76%), and 1.15% export increase. The terms-of-trade improvements likely originate from quality certification premium mechanisms. Given its production of high-quality dairy and meat products, scholars suggest New Zealand may trigger trade diversion effects within the region (Liu and Wang, 2024), though further research is needed to examine its impact on China’s domestic industries and associated technology transfer phenomena. [16]
3. **Australia:** Under the short-term comprehensive policy (S2), Australia’s GDP growth rate is only 0.05%, while its terms of trade improved by 1.98%, with import volume growing by 3.17% and export volume increasing by 5.11%. Under the long-term comprehensive policy (S4), Australia achieved 0.88% GDP growth, 4.13% improvement in terms of trade, 0.98% import growth, and 1.68% export expansion. The minimal short-term growth may validate the resource curse hypothesis [73], where the high proportion of beef exports suppressed TFP growth in the horticulture sector. As a major beef exporter, Australia’s long-term (S4) improvements likely benefited from reduced loss rates due to RCEP’s mutual recognition standards for live animal transportation. Other scholars similarly acknowledge Australia’s significant trade potential with China [24].
4. **ASEAN:** Under the short-term comprehensive policy (S2), ASEAN’s GDP grows by 0.69%, trade conditions improve by 2.99%, import volume grows by 0.91%, and export volume grows by 1.64%. Under the long-term comprehensive policy (S4), ASEAN’s GDP growth rate is 0.75%, trade conditions improve by 8.88%, import volume grows by 0.41%, and export volume increases to 0.63%. The simulation results for ASEAN members may derive from multiple mechanisms: Malaysia’s palm oil refining sector import growth potentially boosted export value-added, thereby promoting GDP growth (supporting the “Factory Asia” hypothesis) [74]; conversely, Thailand’s agricultural processing sectors (e.g., rice) may face unbroken technological bottlenecks, causing export deceleration (reflecting a “middle-technology trap”) [75]. While scholars generally acknowledge RCEP’s significant opportunities for ASEAN, most analyses remain limited to superficial outcome interpretations rather than in-depth causal investigations.
5. **Japan.** Under the short-term comprehensive policy (S2), Japan’s GDP grows by 0.27%, trade conditions deteriorate by 2.63%, import volume grows by 0.92%, and export volume grows by 15.34%. The long-term comprehensive policy (S4) yielded 0.96% GDP growth, 0.50% terms-of-trade improvement, 0.21% import growth, and 1.47% export increase. The short-term dynamics demonstrate Japan’s significant gains from tariff-induced export expansion effects. However, the terms-of-trade deterioration reveals import price declines adversely impacting domestic producers. This phenomenon may stem from either: (a) geographical indication protections under RCEP amplifying national competitive advantages (e.g., Shizuoka green tea) [56], or (b) zombie firm effects (exemplified by Japan’s small-scale rice farmers exhibit merely 3% exit rates versus China’s 23%) [76]. The long-term outcomes reflect how non-tariff barrier reductions substantially improved Japan’s terms of trade through technology diffusion and institutional coordination, while export structure optimization (e.g., increased high-tech product shares) further enhanced international competitiveness. Given Japan and Korea’s strong demand for premium agricultural products, scholars project they will become major export destinations for China’s processed foods and horticultural products [7].
6. **South Korea**. Under the short-term comprehensive policy (S2), South Korea achieved 0.16% GDP growth with 2.51% deterioration in terms of trade, alongside 6.13% import growth and 3.31% export expansion. The long-term comprehensive policy (S4) yielded 0.30% GDP growth, 7.44% terms-of-trade improvement, 3.47% import growth, and 0.77% export increase. The short-term performance reflects South Korea’s initial benefits from tariff-induced export growth, while import price declines negatively impacted domestic producers. Long-term non-tariff barrier reductions enhanced South Korea’s terms of trade and strengthened international competitiveness. The export acceleration may also derive from short-term follower advantages under RCEP [77], exemplified by improved kimchi fermentation efficiency and shortened production cycles, whereas the deceleration aligns with modular bottleneck theory-such as continued reliance on imported core sterilization modules for food processing [78].
7. **Other Countries**. Under the short-term comprehensive policy (S2), non-RCEP countries experienced −0.13% GDP growth, 1.48% terms-of-trade deterioration, 0.59% import contraction, and 2.89% export growth. The long-term comprehensive policy (S4) resulted in −0.02% GDP growth, 1.10% terms-of-trade deterioration, 0.33% import contraction, and 0.85% export growth for non-RCEP members. The short-term outcomes demonstrate negative trade diversion effects on non-member states. Although long-term non-tariff barrier reductions partially mitigated these adverse impacts, trade diversion effects remained significant. Bown’s (2020) circumvention trade strategy theory provides supplementary explanation-for instance, Indonesia replacing the EU as a primary palm oil supplier, or export countries adopting circumvention strategies to bypass rules of origin, like Brazilian soybeans transshipped through Vietnam [79]. Most scholars anticipate trade diversion under RCEP mechanisms [16] alongside supply chain integration [80].

### 5.5. Differential impacts of policy combinations

The policy combination effects under the RCEP framework exhibit significant spatiotemporal heterogeneity, rooted in the interaction of policy instrument attributes and industrial characteristics. Unlike previous studies that simplistically examined tariff reductions or non-tariff factors in isolation, this section establishes a dual-dimensional dynamic analysis framework while systematically investigating the profound impacts of non-tariff barriers on transaction costs, technology spillovers, and industrial organization. This section reveals the internal logic of these differential impacts through theory-data mapping. See Tables 5 and 6.

(1) Asymmetry Between Short-Term and Long-Term Effects. In the short term, pure tariff reductions (S1) have limited effects on economic growth and trade conditions, while the inclusion of non-tariff barrier reductions (S2) significantly improve both. This indicates that non-tariff barrier reductions play a more prominent role in optimizing supply chains and facilitate trade in the short term. In the long term, the effects of pure tariff reductions (S3) are weaker than those from the short-term comprehensive policy (S2). However, the long-term comprehensive policy (S4) significantly enhances economic growth and trade conditions through the dynamic cumulative effects of technology diffusion and institutional coordination.
(2) Synergistic Pathways of Tariff and non-tariff barrier reductions. Tariff concessions directly promote agricultural trade by reducing import prices and enhancing export competitiveness. However, their static effects are partially offset over time by the costs of reallocating production factors, which is consistent with the law of diminishing returns. Non-tariff barrier reductions, on the other hand, achieve price advantages by reducing transaction costs, creating quality premiums, and accelerating technology diffusion. These measures mitigate the pressure of deteriorating trade conditions and promote total factor productivity (TFP) growth. Additionally, shortening inspection and quarantine times, improving customs efficiency, and unifying technical standards significantly reduce trade costs, enhancing the timeliness and competitiveness of agricultural products. Finally, long-term non-tariff barrier reductions further amplify their policy effects through the dynamic cumulative effects of technology diffusion and institutional coordination.
(3) Structural Roots of Sectoral Heterogeneity. Grain production faces short-term impacts from low-priced imports but gradually improves its competitiveness in the long term through technology adoption and scale operations, validating Aghion’s (2018) conclusion on competition-driven innovation. The fruit and vegetable sector benefits in the short term from simplified export inspection and quarantine procedures but may experience slower growth in the long term due to lagging logistics infrastructure upgrades or constraints imposed by transportation costs, which keep loss rates at a fixed level. The dairy sector benefits in the short term from expanded market access due to non-tariff barrier reductions but experiences a slowdown in growth in the long term due to the development of domestic substitutes. The aquatic product sector benefits in the short term from the price competitiveness brought by tariff reductions but sees a decline in import demand in the long term due to the enhanced competitiveness of the domestic aquaculture.

## 6. Conclusions and policy recommendations

### 6.1. Conclusions

Using the GTAT model, this study systematically analyzes the impact of RCEP on agricultural trade among member countries from the perspectives of tariff concessions and non-tariff barrier reductions, and compares the effects of different policy combinations. The main conclusions are as follows:

#### (1) The dominant role of non-tariff barrier reductions.

The institutional openness under the RCEP framework exhibits a significant “dual-wheel drive” characteristic: tariff concessions reshape trade flows through price transmission mechanisms, significantly reducing trade costs for agricultural products among member countries and promoting intra-regional circulation. Non-tariff barrier reductions, on the other hand, optimize resource allocation efficiency through transaction cost channels, significantly enhancing trade facilitation and driving agricultural trade growth.

Under the long-term comprehensive policy scenario (S4), China’s GDP grows by 1.93%, with non-tariff barrier reductions contributing 68.4%. This finding challenges the traditional “tariff dominance” paradigm in trade policy analysis, highlighting the critical role of institutional coordination in deep regional integration.

#### (2) Differentiated responses of agricultural sectors.

The structural differentiation in agricultural trade underscores the complex interplay between policy tools and industrial characteristics. First, sensitive sectors face adjustment pressures. For tariff-sensitive sectors (e.g., dairy, S4: −21.55%), import shocks reveal dual shortcomings in domestic production systems in terms of economies of scale and technical efficiency, but long-term technology imports can lead to output recovery. Second, high-value-added sectors have achieved breakthroughs. The surge in oilseed exports under long-term policies (S4) reflects the role of non-tariff barrier reductions in promoting high-value-added products. Finally, the sharp decline in aquatic product imports (−88.09%, S4) suggests the gradual emergence of the domestic substitution effects, but the modest increase in domestic output(0.61%) indicates potential supply chain restructuring not captured by the model.

#### (3) Differentiated impacts of policy combinations.

The dynamic interaction between tariff and non-tariff measures shows clear asymmetry. In the short term, pure tariff reductions (S1) have limited impact on economic growth and trade conditions, while the inclusion of non-tariff barrier reductions (S2) significantly improves both. In the long term, non-tariff barrier reductions significantly enhance economic growth and trade conditions through the dynamic cumulative effects of technology diffusion and institutional coordination.

Although tariff concessions directly promote agricultural trade by reducing import prices and enhancing export competitiveness, long-term data (S3 → S4) show that the trade creation effects of pure tariff reductions exhibit diminishing returns, while the dynamic gains from institutional coordination continue to emerge. This temporal differentiation suggests that traditional tariff tools have immediate effects in the early stages of regional integration, but sustainable trade dividends depend more on the transaction cost revolution brought by institutional openness.

#### (4) Strategic interaction patterns among member countries.

Member countries exhibit complex strategic interactions with significant heterogeneity. China and ASEAN show strong complementarity in agricultural trade, which RCEP further strengthens; China, Japan, and South Korea compete in capital- and technology-intensive agricultural exports, but RCEP provides opportunities for future agricultural cooperation. Resource-endowed economies (e.g., Australia, New Zealand) gain greater bargaining rents through non-tariff barrier reductions, with RCEP further expanding their market access and export competitiveness. Technology-frontier countries (e.g., Japan) benefit from intellectual property premiums due to mutual recognition of standards. ASEAN exhibits a “low growth, high returns” characteristic in the S4 scenario (GDP + 0.75%, trade conditions +8.88%), likely related to its “hub node” position in regional value chains, capturing industrial chain integration dividends by reducing intermediate trade costs. Lastly, RCEP generates significant trade diversion effects for non-RCEP members, reflecting the complexity and diversity of trade strategies under the RCEP framework.

### 6.2 Policy recommendations

Under the RCEP framework, this paper proposes four strategies regarding the differentiated impacts of tariff and non-tariff policies on the agricultural sector.

(1) Deepening Institutional Opening and Strengthening Collaborative Governance of Non-Tariff Barriers. Advancing Mutual Recognition of Agricultural Product Standards and Innovation in Customs Clearance Mechanisms. Establish a dynamic collaborative mechanism for agricultural quality standards. For sectors significantly impacted in simulations (such as dairy and oilseed sectors), reduce repetitive testing and certification, jointly build a fast customs clearance network to shorten clearance time for time-sensitive products like fruits and vegetables; construct digital trade facilitation infrastructure. Develop a blockchain platform for cross-border agricultural product certification to achieve “one-chain verification and full-domain mutual recognition” among member states; improve policy coordination mechanisms for non-tariff measures, regularly evaluate the implementation effects of SPS (Sanitary and Phytosanitary Measures) and TBT (Technical Barriers to Trade), and promote joint simplification of import licensing procedures by member states.
(2) Implementing Differentiated Policies for the Agricultural Sector to Address Structural Development Bottlenecks. Technical Upgrading and Capacity Optimization for Sensitive Sectors. For tariff-sensitive industries such as dairy products and grains, focus on introducing international advanced technologies to improve production efficiency and enhance buffering capacity against import shocks; carry out value chain extension projects for high-value-added industries, align quality standards of advantageous industries such as oilseeds and fruits/vegetables with international norms, and while enhancing product premium capabilities, foster regional high-value-added agricultural product processing clusters. Supply Chain Reconstruction for Substitute Industries. For sectors showing import substitution trends (e.g., aquatic products), strengthen R&D and promotion of new technologies, explore innovative aquaculture models, and enhance supply chain autonomy. Meanwhile, establish a resilience evaluation system for agricultural supply chains to dynamically track international market fluctuations, and prevent supply chain risks through cross-regional capacity allocation, reserve facility construction, and other means.
(3) Constructing a Dual-Track Policy System of “Short-Term Tariff Regulation–Long-Term Institutional Coordination”. Implement short-term tariff elasticity design, adopting a “category-specific and cycle-specific” tariff adjustment strategy based on the price elasticity of different product categories and domestic industrial competitiveness. Meanwhile, innovate tariff reduction forms and explore new tax reduction models to mitigate the impact of pure tariff cuts on sensitive industries; Second, carry out long-term institutional coordination capacity-building. Formulate an institutional opening plan for RCEP agricultural products, advance rule coordination in technical standards, data flow, etc., in phases, and focus on breaking through bottlenecks such as mutual recognition of inspection and quarantine; Finally, evaluate and adjust the effect of policy combinations. An annual audit mechanism can be established to dynamically adjust the pace of tariff concessions and the focus of non-tariff reforms.
(4) Regional Value Chain Coordination Strategies Based on Member States’ Heterogeneity. First, deepen the complementary cooperation in agricultural industry chains between China and ASEAN, establish a cross-border industrial chain system, jointly build joint planting bases in ASEAN’s advantageous fields, and leverage Chinese technology to enhance production capacity; Second, promote the transformation of cooperation in competitive fields among China, Japan, and South Korea. Establish a tripartite joint R&D mechanism in high-end agricultural products, share technical patents and standard achievements, pilot collaborative supervision, and unleash trade potential; Third, strengthen supply chain stabilization mechanisms with resource-endowed economies. Sign relevant product agreements with Australia and New Zealand to clarify price fluctuation ranges for key products such as dairy products, beef, and mutton. Support Chinese enterprises in participating in agricultural infrastructure upgrading in Australia and New Zealand to exchange investment for market access; Finally, enhance the value chain integration capacity of ASEAN’s hub nodes. Relying on RCEP’s regional accumulation rules, attract the transfer of China’s fruit and vegetable processing, grain and oil refining, and other industries to Southeast Asia, and amplify ASEAN’s dividends as a “middle-goods trade hub.”

### 6.3. Limitations and prospects

First, there is the issue of quantification precision for non-tariff barriers (NTB). The current quantification of NTB uses a uniform reduction ratio, failing to distinguish the differential impacts of heterogeneous measures such as technical barriers, quota restrictions, and export subsidies. However, existing models do not account for such differences, leading to ambiguity in evaluating policy effects. Second, the impact values are calculated based on weighted measurements from existing RCEP commitment schedules, but there are national differences in implementation intensity (e.g., sensitive item exemption clauses in some ASEAN countries), and the elasticity of policy implementation caused by geopolitics (such as China-U.S. competition) is not considered, which may lead to deviations between simulation results and actual conditions. Finally, the broad grouping of agricultural sectors obscures the differentiated response mechanisms of sub-divided products (such as traditional Chinese medicinal materials, flowers, and seed industries), and does not distinguish the value chain position differences between primary products and deep-processed products, making it difficult to reveal the structural upgrading paths for high-value-added agricultural products.

To address the issue of quantification precision for non-tariff barriers (NTB), future research could develop an industry-specific and measure-specific quantification framework. By integrating the World Bank’s NTM database with UNCTAD TRAINS policy documents, natural language processing (NLP) techniques can be employed to extract the constraint intensity of specific clauses and establish a mapping matrix, thereby enhancing the accuracy of policy simulations. Second, regarding the problem of impact valuation, a dynamic database for RCEP policy implementation could be constructed, incorporating Bayesian updating algorithms to calibrate tariff impact parameters in real time. Finally, future studies may focus on characteristic or key agricultural trade products, adopting more granular sectoral and regional classifications to illustrate RCEP’s trade-restructuring effects on these sectors.

## Supporting information

S1 File The customs duty calculation process. (ZIP)

S2 File Code and Results. (DOCX)

S3 File GTAP-Dynamic GTAP Model Unveiling the Temporal Transmission of Policies and the Dynamic Evolution of the Economy. (DOCX)

S4 File Table 1, 2. (XLSX)

S5 File Reporting of Results. (XLSX)

S6 File Econ Map_3.1_ssp5_20240722. (XLSX)

S7 File CEPII Summary Table. (XLSX)

## Abbreviations

RCEP: the Regional Comprehensive Economic Partnership
GTAP: global trade analysis project
SPS: sanitary and phytosanitary measures
TBT: technical barriers to trade
CES: constant elasticity of substitution
CET: constant elasticity of transformation
TFP: total factor productivity
