# Supplementary material for: Impacts of RCEP’s trade barrier reductions on China’s agricultural trade: A GTAP simulation
Source: PLoS One. 2025 Jul 10;20(7):e0328060. doi: 10.1371/journal.pone.0328060 (PMC12244828; doi:10.1371/journal.pone.0328060)
Supplement: S2 File — (DOCX) [file pone.0328060.s002.docx]

Analysis of the Dynamic Impact of the RCEP Entry into Force on the Macroeconomics and Agricultural Trade of Member Countries

1. Basic Information

| No. | String |
| --- | --- |
| 1 | CHN |
| 2 | NZL |
| 3 | AUS |
| 4 | SEAsia |
| 5 | JPN |
| 6 | KOR |
| 7 | RestofWorld |

table1 country and region

| No. | String |
| --- | --- |
| 1 | Cereals |
| 2 | Fruit |
| 3 | Suger |
| 4 | Olis |
| 5 | Animals |
| 6 | Milk |
| 7 | Fishing |
| 8 | BandT |
| 9 | Others |
| 10 | OthServices |

table2 sectors

Database Version: GTAP V10
Solution Method: Euler 50-step Method
Model Used: GTAP-Dyn
Baseline Scenario: There are no trade preferences among countries. The exogenous shock variables are based on the CEPII's forecast data for population, skilled labor, unskilled labor, capital, and GDP. Through the simulation of the baseline scenario, we can obtain the macroeconomic context from 2014 to 2060.
Alternative Scenario: The alternative scenario uses the macroeconomic closure of the policy scenario but applies the variables from the baseline scenario to impact the macroeconomic results.
Policy Scenario: See Table 3.

| S1 | Tariff Reductions, 2022-2025 |
| --- | --- |
| S2 | Reductions in Tariffs and Non-Tariff Trade Barriers, 2022-2025 |
| S3 | Tariff Reductions, 2022-2035 |
| S3 | Reductions in Tariffs and Non-Tariff Trade Barriers, 2022-2035 |

table3 policy

Different policy combinations (tariff and non-tariff barrier reductions) and time dimensions (short-term and long-term) have differentiated impacts on the macroeconomy and agricultural trade.

From the perspective of macroeconomic growth effects, short-term tariff reduction (S1) has a relatively limited impact on China's GDP (0.21%), which reflects that the reduction of tariff barriers alone may face the mutual offset of import substitution and export expansion effects in the short term. When the policy is extended to non-tariff barrier reductions (S2), China's GDP growth rate jumps to 0.78%, indicating that non-tariff measures such as improved customs clearance efficiency and mutual recognition of technical standards have a more significant role in optimizing supply chains, especially in shortening the cross-border logistics cycle of agricultural products. In the long-term scenario, the effect of sole tariff reduction (S3) (0.24%) is weaker than that of the short-term comprehensive policy (S2), which may be due to the static effect of tariff reduction being partially offset by the cost of reconfiguration of production factors over time. However, under the long-term comprehensive policy (S4), China's GDP growth rate reaches 1.93%, highlighting the dynamic cumulative effect of non-tariff barrier reductions through technology diffusion and institutional coordination. For example, the long-term convergence of agricultural quality certification systems reduces the cost of repeated inspections, while the unification of cross-border e-commerce rules continues to release the benefits of trade facilitation.

The dynamic changes in the terms of trade (TOT) reveal the distribution effects of policy combinations. Under short-term tariff reduction (S1), China's terms of trade deteriorate by 3.14%, mainly because the tariff concessions lead to a relative decline in the price of imported goods, while the price of exported goods is constrained by international market competition and cannot be adjusted synchronously. When non-tariff barrier reductions are included (S2), the degree of deterioration in the terms of trade narrows to 1.54%, indicating that the reduction in customs clearance costs allows exporters to maintain a higher free-on-board price. In the long-term scenario, the degree of deterioration in the terms of trade in the S3 phase (-2.05%) exceeds that of S1, possibly reflecting that sustained tariff reductions have intensified price competition in the export market. In the S4 phase, the degree of deterioration further narrows to -1.54%, confirming that non-tariff barrier reductions partially offset downward price pressure by increasing the added value of exported goods (e.g., the premium capacity of organic certified agricultural products). Resource-based economies such as New Zealand and Australia see an improvement in their terms of trade by 5.05% and 4.13% in the S4 phase, respectively, showing that they gain greater bargaining advantages in non-tariff barrier reductions with their long-established quality traceability systems, especially the differentiated positioning of dairy and beef products that makes them immune to price competition shocks.

The agricultural output effects show significant sectoral heterogeneity and policy dependence. China's grain production shrinks by 3.54% under the short-term comprehensive policy (S2), mainly due to the combined impact of tariff and non-tariff barrier reductions on low-priced imported wheat. However, under the long-term comprehensive policy (S4), output recovers to 0.29%, indicating that domestic producers gradually enhance their competitiveness through technology adoption (e.g., the promotion of stress-resistant varieties) and large-scale operations. The fruit and vegetable sector maintains a growth rate of 2.71% and 1.47% in the short-term policies (S1-S2), benefiting from the expansion of international market access due to the simplification of export inspection and quarantine procedures. However, in the long term (S3-S4), the growth rate slows down to 0.09% and -0.29%, respectively, revealing the bottleneck of loss rate caused by the lag in the upgrading of fresh agricultural product logistics infrastructure. New Zealand's dairy output surges by 9.52% in the S2 phase, confirming the key role of non-tariff barrier reductions in its high-value whey protein exports—breaking through the EU's geographical indication protection system allows it to quickly capture the Asian health product market. Japan's grain output abnormally increases by 22.76% and 26.18% in the S1-S2 phases, essentially reflecting the reactive strengthening of domestic price support policies under short-term policy shocks. However, in the S3-S4 phases, output falls back to -0.85% and -0.90%, respectively, revealing the unsustainable production expansion under land resource constraints.

In terms of import trade effects, China's agricultural imports soar by 10.20% under the short-term comprehensive policy (S2), with the import elasticity coefficient of dairy products as high as 1.32, showing that non-tariff barrier reductions (e.g., mutual recognition of infant formula registration systems) significantly lower the market entry threshold for high-quality products. However, under the long-term comprehensive policy (S4), the import growth rate falls back to 5.16%, reflecting the development of domestic substitutes (e.g., the expansion of domestic A2 beta-casein milk production capacity) and rationalization of consumption preferences. ASEAN's import growth is 0.91% in the S2 phase, mainly due to the increase in intermediate product trade brought about by the simplification of China-ASEAN rules of origin, such as Malaysia's import of palm oil refining equipment for processing and re-export. South Korea's imports contract by -0.33% in the S4 phase, which is related to its "positive list" system established in the long-term non-tariff barrier reductions, achieving selective import substitution through strengthened technical barriers such as GMO labeling.

Export trade effects demonstrate the multiplier effect of policy instruments. China's exports grow by 4.84% in the S2 phase, with frozen fruit and vegetable exports increasing by 12.7%, directly benefiting from the transparency of sanitary and phytosanitary (SPS) measures in the target market, which reduces compliance costs. However, under the long-term comprehensive policy (S4), the export growth rate drops to 0.91%, indicating intensified competition from latecomer countries with similar products, such as Vietnam's lychees squeezing the export share of fresh fruit in South China with lower customs clearance costs. New Zealand's dairy exports grow by 7.95% in the S4 phase, reflecting its deep market penetration using the long-established non-tariff barrier recognition network (e.g., the upgraded free trade agreement with China). Australia's sugar exports maintain a growth rate of 2.26%-2.55% in the S3-S4 phases, reflecting its extension of the tariff reduction effect to the regional value chain through the rules of origin accumulation, such as importing Brazilian raw sugar, refining it in Australia, and entering China tariff-free. ASEAN's fisheries exports contract significantly by 12.61% in the S4 phase, exposing the failure of its aquaculture sector to meet the quality traceability standards required by long-term non-tariff barrier reductions, such as the lack of EU IUU (Illegal, Unreported, and Unregulated) certification leading to order diversion.

Code（S1）

*! Begin CMFSTART file***servants**=1;
**start with** **MMNZ** = 40000000 ;

*! End CMFSTART file*
**auxiliary** **files** = .\CODE\GTAP;
**Solution** **file** = .\s-1 ;
**Method** = **euler**;
**Steps** = 200;

**accuracy** **percent** = 95;
**verbal** **description** = .\autosim rr-17_21;
**File** GTAPSETS = .\**DATA**\**sets**.har; *! constant input***File** GTAPDATA = dat-22_25.har; *! updated input***File** GTAPPARM = .\**DATA**\**default**.prm ; *! constant input***updated** **file** GTAPDATA = .\dat-s-1.har;
**intermediate** **extra** **data** =.\s-1 ;
**log file**=.\s-1.log;
**CPU**=**YES**;

*!neq= yes; !use if you want to check for errors in tab and cmf files!
!but dont wanna involve the data and calculations!
! Begin Common Closure file***Exogenous**
 pop
 psaveslack pfactwld
 profitslack incomeslack endwslack
 cgdslack tradslack
 ams atm atf ats atd
 aosec aoreg avasec avareg
 afcom afsec afreg afecom afesec afereg
 afall afeall aoall
 au dppriv dpgov dpsave
 **to** tp tm tms tx txs
 qo(ENDW_COMM,REG);

**Rest** **endogenous**;

*!swap aoreg(reg)=qgdp(reg);*
*! End Common Closure file*
*! The BASE Case MACRO Shocks
! --------------------
!*
*! Begin Period-Specific Shocks file
!==== Macro shocks ============================================================!***shock** POP(reg) = **select** **from** **file** POPCEPII_1.HAR **header** *"GROW"* slice *"Y2025"* ;
*!shock qgdp(reg) = select from file GDPCEPII.HAR header "GROW" slice "Y2027";***shock** qo(*"unsklab"*,reg) = **select** **from** **file** UNSKLSHKLI_1.HAR **header** *"YR25"* ;
**shock** qo(*"sklab"*,reg) = **select** **from** **file** SKLSHKLI_1.HAR **header** *"YR25"*;
**shock** qo(*"Capital"*,reg) = **select** **from** **file** CAPSHKLi_1.HAR **header** *"YR25"* ;
*!============================================================================!*
**shock** qo(*"natres"*,reg)=**uniform** 3.1079601; *!.7% per years, with exponentional growth
!1 0.7
!2 1.449
!3 2.25043
!4 3.1079601*
*! End Period-Specific Shocks file*
*!shock ams(Trad_comm, "CHINA", "USA") = uniform 1;***Shock** tms(*"Cereals"*,*"NZL"*,*"CHN"*) = -0.026;
 **Shock** tms(*"Fruit"*,*"NZL"*,*"CHN"*) = -0.112;
 **Shock** tms(*"Suger"*,*"NZL"*,*"CHN"*) = -0.014;
 **Shock** tms(*"Olis"*,*"NZL"*,*"CHN"*) = 0.000;
 **Shock** tms(*"Animals"*,*"NZL"*,*"CHN"*) = -0.113;
 **Shock** tms(*"Milk"*,*"NZL"*,*"CHN"*) = -0.004;
 **Shock** tms(*"Fishing"*,*"NZL"*,*"CHN"*) = 0.0000;
 **Shock** tms(*"BandT"*,*"NZL"*,*"CHN"*) = 0.0000;
 **Shock** tms(*"Others"*,*"NZL"*,*"CHN"*) = -0.0000;
 **Shock** tms(*"Cereals"*,*"AUS"*,*"CHN"*) = -0.012;
 **Shock** tms(*"Fruit"*,*"AUS"*,*"CHN"*) = -0.988;
 **Shock** tms(*"Suger"*,*"AUS"*,*"CHN"*) = -0.648;
 **Shock** tms(*"Olis"*,*"AUS"*,*"CHN"*) = -0.337;
 **Shock** tms(*"Animals"*,*"AUS"*,*"CHN"*) = -0.199;
 **Shock** tms(*"Milk"*,*"AUS"*,*"CHN"*) = -0.019;
 **Shock** tms(*"Fishing"*,*"AUS"*,*"CHN"*) = -0.541;
 **Shock** tms(*"BandT"*,*"AUS"*,*"CHN"*) = -0.889;
 **Shock** tms(*"Others"*,*"AUS"*,*"CHN"*) = -0.192;
 **Shock** tms(*"Cereals"*,*"SEAsia"*,*"CHN"*) = -0.035;
 **Shock** tms(*"Fruit"*,*"SEAsia"*,*"CHN"*) = -0.0000;
 **Shock** tms(*"Suger"*,*"SEAsia"*,*"CHN"*) = -0.751;
 **Shock** tms(*"Olis"*,*"SEAsia"*,*"CHN"*) = -0.068;
 **Shock** tms(*"Animals"*,*"SEAsia"*,*"CHN"*) = 0.000;
 **Shock** tms(*"Milk"*,*"SEAsia"*,*"CHN"*) = -0.018;
 **Shock** tms(*"Fishing"*,*"SEAsia"*,*"CHN"*) = 0.0000;
 **Shock** tms(*"BandT"*,*"SEAsia"*,*"CHN"*) = -0.224;
 **Shock** tms(*"Others"*,*"SEAsia"*,*"CHN"*) = -0.035;
 **Shock** tms(*"Cereals"*,*"JPN"*,*"CHN"*) = -0.015;
 **Shock** tms(*"Fruit"*,*"JPN"*,*"CHN"*) = -0.055;
 **Shock** tms(*"Suger"*,*"JPN"*,*"CHN"*) = 0.00;
 **Shock** tms(*"Olis"*,*"JPN"*,*"CHN"*) = -0.344;
 **Shock** tms(*"Animals"*,*"JPN"*,*"CHN"*) = -0.140;
 **Shock** tms(*"Milk"*,*"JPN"*,*"CHN"*) = -0.395;
 **Shock** tms(*"Fishing"*,*"JPN"*,*"CHN"*) = -0.131;
 **Shock** tms(*"BandT"*,*"JPN"*,*"CHN"*) = -0.221;
 **Shock** tms(*"Others"*,*"JPN"*,*"CHN"*) = -0.469;
 **Shock** tms(*"Cereals"*,*"KOR"*,*"CHN"*) = -0.033;
 **Shock** tms(*"Fruit"*,*"KOR"*,*"CHN"*) = -0.604;
 **Shock** tms(*"Suger"*,*"KOR"*,*"CHN"*) = 0.0000;
 **Shock** tms(*"Olis"*,*"KOR"*,*"CHN"*) = -0.397;
 **Shock** tms(*"Animals"*,*"KOR"*,*"CHN"*) = -0.395;
 **Shock** tms(*"Milk"*,*"KOR"*,*"CHN"*) = -0.097;
 **Shock** tms(*"Fishing"*,*"KOR"*,*"CHN"*) = -0.261;
 **Shock** tms(*"BandT"*,*"KOR"*,*"CHN"*) = -0.481;
 **Shock** tms(*"Others"*,*"KOR"*,*"CHN"*) = -0.398;
**Shock** tms(*"Cereals"*,*"CHN"*,*"AUS"*) = 0.0000;
 **Shock** tms(*"Fruit"*,*"CHN"*,*"AUS"*) = -0.1277;
 **Shock** tms(*"Suger"*,*"CHN"*,*"AUS"*) = -2.9521;
 **Shock** tms(*"Olis"*,*"CHN"*,*"AUS"*) = -0.1404;
 **Shock** tms(*"Animals"*,*"CHN"*,*"AUS"*) = -0.5113;
 **Shock** tms(*"Milk"*,*"CHN"*,*"AUS"*) = 0.0000;
 **Shock** tms(*"Fishing"*,*"CHN"*,*"AUS"*) = 0.0000;
 **Shock** tms(*"BandT"*,*"CHN"*,*"AUS"*) = -0.8502;
 **Shock** tms(*"Others"*,*"CHN"*,*"AUS"*) = -1.5847;
 **Shock** tms(*"Cereals"*,*"CHN"*,*"JPN"*) = -1.1684;
 **Shock** tms(*"Fruit"*,*"CHN"*,*"JPN"*) = -1.0852;
 **Shock** tms(*"Suger"*,*"CHN"*,*"JPN"*) = -11.4830;
 **Shock** tms(*"Olis"*,*"CHN"*,*"JPN"*) = -0.0824;
 **Shock** tms(*"Animals"*,*"CHN"*,*"JPN"*) = -1.7696;
 **Shock** tms(*"Milk"*,*"CHN"*,*"JPN"*) = 0.0000;
 **Shock** tms(*"Fishing"*,*"CHN"*,*"JPN"*) = -0.9383;
 **Shock** tms(*"BandT"*,*"CHN"*,*"JPN"*) = -1.2304;
 **Shock** tms(*"Others"*,*"CHN"*,*"JPN"*) = -1.6081;
 **Shock** tms(*"Cereals"*,*"CHN"*,*"KOR"*) = -7.0914;
 **Shock** tms(*"Fruit"*,*"CHN"*,*"KOR"*) = -40.1571;
 **Shock** tms(*"Suger"*,*"CHN"*,*"KOR"*) = -24.7656;
 **Shock** tms(*"Olis"*,*"CHN"*,*"KOR"*) = -62.9511;
 **Shock** tms(*"Animals"*,*"CHN"*,*"KOR"*) = -4.2102;
 **Shock** tms(*"Milk"*,*"CHN"*,*"KOR"*) = -25.7707;
 **Shock** tms(*"Fishing"*,*"CHN"*,*"KOR"*) = -4.8948;
 **Shock** tms(*"BandT"*,*"CHN"*,*"KOR"*) = -13.0734;
 **Shock** tms(*"Others"*,*"CHN"*,*"KOR"*) = -18.1884;
 **Shock** tms(*"Cereals"*,*"CHN"*,*"SEAsia"*) = -3.0166;
 **Shock** tms(*"Fruit"*,*"CHN"*,*"SEAsia"*) = -0.4503;
 **Shock** tms(*"Suger"*,*"CHN"*,*"SEAsia"*) = -0.3837;
 **Shock** tms(*"Olis"*,*"CHN"*,*"SEAsia"*) = -0.5135;
 **Shock** tms(*"Animals"*,*"CHN"*,*"SEAsia"*) = -1.4898;
 **Shock** tms(*"Milk"*,*"CHN"*,*"SEAsia"*) = 0.0000;
 **Shock** tms(*"Fishing"*,*"CHN"*,*"SEAsia"*) = -0.0013;
 **Shock** tms(*"BandT"*,*"CHN"*,*"SEAsia"*) = -9.6385;
 **Shock** tms(*"Others"*,*"CHN"*,*"SEAsia"*) = -1.4473;
 **Shock** tms(*"Cereals"*,*"CHN"*,*"NZL"*) = 0.0000;
 **Shock** tms(*"Fruit"*,*"CHN"*,*"NZL"*) = 0.0000;
 **Shock** tms(*"Suger"*,*"CHN"*,*"NZL"*) = 0.0000;
 **Shock** tms(*"Olis"*,*"CHN"*,*"NZL"*) = 0.0000;
 **Shock** tms(*"Animals"*,*"CHN"*,*"NZL"*) = 0.0000;
 **Shock** tms(*"Milk"*,*"CHN"*,*"NZL"*) = 0.0000;
 **Shock** tms(*"Fishing"*,*"CHN"*,*"NZL"*) = 0.0000;
 **Shock** tms(*"BandT"*,*"CHN"*,*"NZL"*) = 0.0000;
 **Shock** tms(*"Others"*,*"CHN"*,*"NZL"*) = 0.0000;
S2

*! Begin CMFSTART file***servants**=1;
**start with** **MMNZ** = 40000000 ;

*! End CMFSTART file*
**auxiliary** **files** = .\CODE\GTAP;
**Solution** **file** = .\s-2 ;
**Method** = **euler**;
**Steps** = 50;

**accuracy** **percent** = 90;
**verbal** **description** = .\autosim rr-17_21;
**File** GTAPSETS = .\**DATA**\**sets**.har; *! constant input***File** GTAPDATA = dat-22_25.har; *! updated input***File** GTAPPARM = .\**DATA**\**default**.prm ; *! constant input***updated** **file** GTAPDATA = .\dat-s-2.har;
**intermediate** **extra** **data** =.\s-2 ;
**log file**=.\s-2.log;
**CPU**=**YES**;

*!neq= yes; !use if you want to check for errors in tab and cmf files!
!but dont wanna involve the data and calculations!
! Begin Common Closure file***Exogenous**
 pop
 psaveslack pfactwld
 profitslack incomeslack endwslack
 cgdslack tradslack
 ams atm atf ats atd
 aosec aoreg avasec avareg
 afcom afsec afreg afecom afesec afereg
 afall afeall aoall
 au dppriv dpgov dpsave
 **to** tp tm tms tx txs
 qo(ENDW_COMM,REG);

**Rest** **endogenous**;

*!swap aoreg(reg)=qgdp(reg);*
*! End Common Closure file*
*! The BASE Case MACRO Shocks
! --------------------
!*
*! Begin Period-Specific Shocks file
!==== Macro shocks ============================================================!***shock** POP(reg) = **select** **from** **file** POPCEPII_1.HAR **header** *"GROW"* slice *"Y2025"* ;
*!shock qgdp(reg) = select from file GDPCEPII.HAR header "GROW" slice "Y2027";***shock** qo(*"unsklab"*,reg) = **select** **from** **file** UNSKLSHKLI_1.HAR **header** *"YR25"* ;
**shock** qo(*"sklab"*,reg) = **select** **from** **file** SKLSHKLI_1.HAR **header** *"YR25"*;
**shock** qo(*"Capital"*,reg) = **select** **from** **file** CAPSHKLi_1.HAR **header** *"YR25"* ;
*!============================================================================!*
**shock** qo(*"natres"*,reg)=**uniform** 3.1079601; *!.7% per years, with exponentional growth
!1 0.7
!2 1.449
!3 2.25043
!4 3.1079601*
*! End Period-Specific Shocks file*


**Shock** ams(TRAD_COMM,REG,*"CHN"*) = **uniform** 2;

**Shock** tms(*"Cereals"*,*"NZL"*,*"CHN"*) = -0.026;
 **Shock** tms(*"Fruit"*,*"NZL"*,*"CHN"*) = -0.112;
 **Shock** tms(*"Suger"*,*"NZL"*,*"CHN"*) = -0.014;
 **Shock** tms(*"Olis"*,*"NZL"*,*"CHN"*) = 0.000;
 **Shock** tms(*"Animals"*,*"NZL"*,*"CHN"*) = -0.113;
 **Shock** tms(*"Milk"*,*"NZL"*,*"CHN"*) = -0.004;
 **Shock** tms(*"Fishing"*,*"NZL"*,*"CHN"*) = 0.0000;
 **Shock** tms(*"BandT"*,*"NZL"*,*"CHN"*) = 0.0000;
 **Shock** tms(*"Others"*,*"NZL"*,*"CHN"*) = -0.0000;
 **Shock** tms(*"Cereals"*,*"AUS"*,*"CHN"*) = -0.012;
 **Shock** tms(*"Fruit"*,*"AUS"*,*"CHN"*) = -0.988;
 **Shock** tms(*"Suger"*,*"AUS"*,*"CHN"*) = -0.648;
 **Shock** tms(*"Olis"*,*"AUS"*,*"CHN"*) = -0.337;
 **Shock** tms(*"Animals"*,*"AUS"*,*"CHN"*) = -0.199;
 **Shock** tms(*"Milk"*,*"AUS"*,*"CHN"*) = -0.019;
 **Shock** tms(*"Fishing"*,*"AUS"*,*"CHN"*) = -0.541;
 **Shock** tms(*"BandT"*,*"AUS"*,*"CHN"*) = -0.889;
 **Shock** tms(*"Others"*,*"AUS"*,*"CHN"*) = -0.192;
 **Shock** tms(*"Cereals"*,*"SEAsia"*,*"CHN"*) = -0.035;
 **Shock** tms(*"Fruit"*,*"SEAsia"*,*"CHN"*) = -0.0000;
 **Shock** tms(*"Suger"*,*"SEAsia"*,*"CHN"*) = -0.751;
 **Shock** tms(*"Olis"*,*"SEAsia"*,*"CHN"*) = -0.068;
 **Shock** tms(*"Animals"*,*"SEAsia"*,*"CHN"*) = 0.000;
 **Shock** tms(*"Milk"*,*"SEAsia"*,*"CHN"*) = -0.018;
 **Shock** tms(*"Fishing"*,*"SEAsia"*,*"CHN"*) = 0.0000;
 **Shock** tms(*"BandT"*,*"SEAsia"*,*"CHN"*) = -0.224;
 **Shock** tms(*"Others"*,*"SEAsia"*,*"CHN"*) = -0.035;
 **Shock** tms(*"Cereals"*,*"JPN"*,*"CHN"*) = -0.015;
 **Shock** tms(*"Fruit"*,*"JPN"*,*"CHN"*) = -0.055;
 **Shock** tms(*"Suger"*,*"JPN"*,*"CHN"*) = 0.00;
 **Shock** tms(*"Olis"*,*"JPN"*,*"CHN"*) = -0.344;
 **Shock** tms(*"Animals"*,*"JPN"*,*"CHN"*) = -0.140;
 **Shock** tms(*"Milk"*,*"JPN"*,*"CHN"*) = -0.395;
 **Shock** tms(*"Fishing"*,*"JPN"*,*"CHN"*) = -0.131;
 **Shock** tms(*"BandT"*,*"JPN"*,*"CHN"*) = -0.221;
 **Shock** tms(*"Others"*,*"JPN"*,*"CHN"*) = -0.469;
 **Shock** tms(*"Cereals"*,*"KOR"*,*"CHN"*) = -0.033;
 **Shock** tms(*"Fruit"*,*"KOR"*,*"CHN"*) = -0.604;
 **Shock** tms(*"Suger"*,*"KOR"*,*"CHN"*) = 0.0000;
 **Shock** tms(*"Olis"*,*"KOR"*,*"CHN"*) = -0.397;
 **Shock** tms(*"Animals"*,*"KOR"*,*"CHN"*) = -0.395;
 **Shock** tms(*"Milk"*,*"KOR"*,*"CHN"*) = -0.097;
 **Shock** tms(*"Fishing"*,*"KOR"*,*"CHN"*) = -0.261;
 **Shock** tms(*"BandT"*,*"KOR"*,*"CHN"*) = -0.481;
 **Shock** tms(*"Others"*,*"KOR"*,*"CHN"*) = -0.398;
**Shock** tms(*"Cereals"*,*"CHN"*,*"AUS"*) = 0.0000;
 **Shock** tms(*"Fruit"*,*"CHN"*,*"AUS"*) = -0.1277;
 **Shock** tms(*"Suger"*,*"CHN"*,*"AUS"*) = -2.9521;
 **Shock** tms(*"Olis"*,*"CHN"*,*"AUS"*) = -0.1404;
 **Shock** tms(*"Animals"*,*"CHN"*,*"AUS"*) = -0.5113;
 **Shock** tms(*"Milk"*,*"CHN"*,*"AUS"*) = 0.0000;
 **Shock** tms(*"Fishing"*,*"CHN"*,*"AUS"*) = 0.0000;
 **Shock** tms(*"BandT"*,*"CHN"*,*"AUS"*) = -0.8502;
 **Shock** tms(*"Others"*,*"CHN"*,*"AUS"*) = -1.5847;
 **Shock** tms(*"Cereals"*,*"CHN"*,*"JPN"*) = -1.1684;
 **Shock** tms(*"Fruit"*,*"CHN"*,*"JPN"*) = -1.0852;
 **Shock** tms(*"Suger"*,*"CHN"*,*"JPN"*) = -11.4830;
 **Shock** tms(*"Olis"*,*"CHN"*,*"JPN"*) = -0.0824;
 **Shock** tms(*"Animals"*,*"CHN"*,*"JPN"*) = -1.7696;
 **Shock** tms(*"Milk"*,*"CHN"*,*"JPN"*) = 0.0000;
 **Shock** tms(*"Fishing"*,*"CHN"*,*"JPN"*) = -0.9383;
 **Shock** tms(*"BandT"*,*"CHN"*,*"JPN"*) = -1.2304;
 **Shock** tms(*"Others"*,*"CHN"*,*"JPN"*) = -1.6081;
 **Shock** tms(*"Cereals"*,*"CHN"*,*"KOR"*) = -7.0914;
 **Shock** tms(*"Fruit"*,*"CHN"*,*"KOR"*) = -40.1571;
 **Shock** tms(*"Suger"*,*"CHN"*,*"KOR"*) = -24.7656;
 **Shock** tms(*"Olis"*,*"CHN"*,*"KOR"*) = -62.9511;
 **Shock** tms(*"Animals"*,*"CHN"*,*"KOR"*) = -4.2102;
 **Shock** tms(*"Milk"*,*"CHN"*,*"KOR"*) = -25.7707;
 **Shock** tms(*"Fishing"*,*"CHN"*,*"KOR"*) = -4.8948;
 **Shock** tms(*"BandT"*,*"CHN"*,*"KOR"*) = -13.0734;
 **Shock** tms(*"Others"*,*"CHN"*,*"KOR"*) = -18.1884;
 **Shock** tms(*"Cereals"*,*"CHN"*,*"SEAsia"*) = -3.0166;
 **Shock** tms(*"Fruit"*,*"CHN"*,*"SEAsia"*) = -0.4503;
 **Shock** tms(*"Suger"*,*"CHN"*,*"SEAsia"*) = -0.3837;
 **Shock** tms(*"Olis"*,*"CHN"*,*"SEAsia"*) = -0.5135;
 **Shock** tms(*"Animals"*,*"CHN"*,*"SEAsia"*) = -1.4898;
 **Shock** tms(*"Milk"*,*"CHN"*,*"SEAsia"*) = 0.0000;
 **Shock** tms(*"Fishing"*,*"CHN"*,*"SEAsia"*) = -0.0013;
 **Shock** tms(*"BandT"*,*"CHN"*,*"SEAsia"*) = -9.6385;
 **Shock** tms(*"Others"*,*"CHN"*,*"SEAsia"*) = -1.4473;
 **Shock** tms(*"Cereals"*,*"CHN"*,*"NZL"*) = 0.0000;
 **Shock** tms(*"Fruit"*,*"CHN"*,*"NZL"*) = 0.0000;
 **Shock** tms(*"Suger"*,*"CHN"*,*"NZL"*) = 0.0000;
 **Shock** tms(*"Olis"*,*"CHN"*,*"NZL"*) = 0.0000;
 **Shock** tms(*"Animals"*,*"CHN"*,*"NZL"*) = 0.0000;
 **Shock** tms(*"Milk"*,*"CHN"*,*"NZL"*) = 0.0000;
 **Shock** tms(*"Fishing"*,*"CHN"*,*"NZL"*) = 0.0000;
 **Shock** tms(*"BandT"*,*"CHN"*,*"NZL"*) = 0.0000;
 **Shock** tms(*"Others"*,*"CHN"*,*"NZL"*) = 0.0000;

S3

*! Begin CMFSTART file***servants**=1;
**start with** **MMNZ** = 40000000 ;

*! End CMFSTART file*
**auxiliary** **files** = .\CODE\GTAP;
**Solution** **file** = .\s-3 ;
**Method** = **euler**;
**Steps** = 50;

**accuracy** **percent** = 90;
**verbal** **description** = .\autosim rr-22_35;
**File** GTAPSETS = .\**DATA**\**sets**.har; *! constant input***File** GTAPDATA = dat-17_21.har; *! updated input***File** GTAPPARM = .\**DATA**\**default**.prm ; *! constant input***updated** **file** GTAPDATA = .\dat-s-3.har;
**intermediate** **extra** **data** =.\s-3 ;
**log file**=.\s-3.log;
**CPU**=**YES**;

*!neq= yes; !use if you want to check for errors in tab and cmf files!
!but dont wanna involve the data and calculations!
! Begin Common Closure file***Exogenous**
 pop
 psaveslack pfactwld
 profitslack incomeslack endwslack
 cgdslack tradslack
 ams atm atf ats atd
 aosec aoreg avasec avareg
 afcom afsec afreg afecom afesec afereg
 afall afeall aoall
 au dppriv dpgov dpsave
 **to** tp tm tms tx txs
 qo(ENDW_COMM,REG);

**Rest** **endogenous**;

*!swap aoreg(reg)=qgdp(reg);*
*! End Common Closure file*
*! The BASE Case MACRO Shocks
! --------------------
!*
*! Begin Period-Specific Shocks file
!==== Macro shocks ============================================================!***shock** POP(reg) = **select** **from** **file** POPCEPII_2.HAR **header** *"GROW"* slice *"Y2035"* ;
*!shock qgdp(reg) = select from file GDPCEPII.HAR header "GROW" slice "Y2027";***shock** qo(*"unsklab"*,reg) = **select** **from** **file** UNSKLSHKLI_2.HAR **header** *"YR35"* ;
**shock** qo(*"sklab"*,reg) = **select** **from** **file** SKLSHKLI_2.HAR **header** *"YR35"*;
**shock** qo(*"Capital"*,reg) = **select** **from** **file** CAPSHKLi_2.HAR **header** *"YR35"* ;
*!============================================================================!*
**shock** qo(*"natres"*,reg)=**uniform** 9.1; *!.7% per years, with exponentional growth
!1 0.7
!2 1.449
!3 2.25043
!4 3.1079601*
*! End Period-Specific Shocks file***Shock** tms(*"Cereals"*,*"NZL"*,*"CHN"*) = -0.2781;
 **Shock** tms(*"Fruit"*,*"NZL"*,*"CHN"*) = -2.6852;
 **Shock** tms(*"Suger"*,*"NZL"*,*"CHN"*) = -0.2062;
 **Shock** tms(*"Olis"*,*"NZL"*,*"CHN"*) = 0.0000;
 **Shock** tms(*"Animals"*,*"NZL"*,*"CHN"*) = -1.6781;
 **Shock** tms(*"Milk"*,*"NZL"*,*"CHN"*) = -1.6126;
 **Shock** tms(*"Fishing"*,*"NZL"*,*"CHN"*) = 0.0000;
 **Shock** tms(*"BandT"*,*"NZL"*,*"CHN"*) = 0.0000;
 **Shock** tms(*"Others"*,*"NZL"*,*"CHN"*) = -0.0001;
 **Shock** tms(*"Cereals"*,*"AUS"*,*"CHN"*) = -0.1870;
 **Shock** tms(*"Fruit"*,*"AUS"*,*"CHN"*) = -10.4354;
 **Shock** tms(*"Suger"*,*"AUS"*,*"CHN"*) = -10.3994;
 **Shock** tms(*"Olis"*,*"AUS"*,*"CHN"*) = -5.0821;
 **Shock** tms(*"Animals"*,*"AUS"*,*"CHN"*) = -3.2376;
 **Shock** tms(*"Milk"*,*"AUS"*,*"CHN"*) = -2.9920;
 **Shock** tms(*"Fishing"*,*"AUS"*,*"CHN"*) = -6.2012;
 **Shock** tms(*"BandT"*,*"AUS"*,*"CHN"*) = -10.2706;
 **Shock** tms(*"Others"*,*"AUS"*,*"CHN"*) = -5.1284;
 **Shock** tms(*"Cereals"*,*"SEAsia"*,*"CHN"*) = -0.4036;
 **Shock** tms(*"Fruit"*,*"SEAsia"*,*"CHN"*) = -0.0001;
 **Shock** tms(*"Suger"*,*"SEAsia"*,*"CHN"*) = -8.0158;
 **Shock** tms(*"Olis"*,*"SEAsia"*,*"CHN"*) = -1.3171;
 **Shock** tms(*"Animals"*,*"SEAsia"*,*"CHN"*) = 0.0000;
 **Shock** tms(*"Milk"*,*"SEAsia"*,*"CHN"*) = -0.2734;
 **Shock** tms(*"Fishing"*,*"SEAsia"*,*"CHN"*) = 0.0000;
 **Shock** tms(*"BandT"*,*"SEAsia"*,*"CHN"*) = -4.4746;
 **Shock** tms(*"Others"*,*"SEAsia"*,*"CHN"*) = -0.6597;
 **Shock** tms(*"Cereals"*,*"JPN"*,*"CHN"*) = -0.4827;
 **Shock** tms(*"Fruit"*,*"JPN"*,*"CHN"*) = -1.7084;
 **Shock** tms(*"Suger"*,*"JPN"*,*"CHN"*) = 0.0000;
 **Shock** tms(*"Olis"*,*"JPN"*,*"CHN"*) = -9.6693;
 **Shock** tms(*"Animals"*,*"JPN"*,*"CHN"*) = -3.7812;
 **Shock** tms(*"Milk"*,*"JPN"*,*"CHN"*) = -13.7500;
 **Shock** tms(*"Fishing"*,*"JPN"*,*"CHN"*) = -3.3648;
 **Shock** tms(*"BandT"*,*"JPN"*,*"CHN"*) = -7.1773;
 **Shock** tms(*"Others"*,*"JPN"*,*"CHN"*) = -7.1347;
 **Shock** tms(*"Cereals"*,*"KOR"*,*"CHN"*) = -0.5741;
 **Shock** tms(*"Fruit"*,*"KOR"*,*"CHN"*) = -11.9383;
 **Shock** tms(*"Suger"*,*"KOR"*,*"CHN"*) = 0.0000;
 **Shock** tms(*"Olis"*,*"KOR"*,*"CHN"*) = -9.9984;
 **Shock** tms(*"Animals"*,*"KOR"*,*"CHN"*) = -8.0651;
 **Shock** tms(*"Milk"*,*"KOR"*,*"CHN"*) = -3.0610;
 **Shock** tms(*"Fishing"*,*"KOR"*,*"CHN"*) = -5.1362;
 **Shock** tms(*"BandT"*,*"KOR"*,*"CHN"*) = -5.3622;
 **Shock** tms(*"Others"*,*"KOR"*,*"CHN"*) = -6.7406;
 **Shock** tms(*"Cereals"*,*"CHN"*,*"AUS"*) = 0.0000;
 **Shock** tms(*"Fruit"*,*"CHN"*,*"AUS"*) = -0.1375;
 **Shock** tms(*"Suger"*,*"CHN"*,*"AUS"*) = -2.9521;
 **Shock** tms(*"Olis"*,*"CHN"*,*"AUS"*) = -0.2780;
 **Shock** tms(*"Animals"*,*"CHN"*,*"AUS"*) = -1.2781;
 **Shock** tms(*"Fishing"*,*"CHN"*,*"AUS"*) = 0.0000;
 **Shock** tms(*"BandT"*,*"CHN"*,*"AUS"*) = -1.0122;
 **Shock** tms(*"Others"*,*"CHN"*,*"AUS"*) = -1.5847;
 **Shock** tms(*"Cereals"*,*"CHN"*,*"JPN"*) = -2.0446;
 **Shock** tms(*"Fruit"*,*"CHN"*,*"JPN"*) = -3.2556;
 **Shock** tms(*"Suger"*,*"CHN"*,*"JPN"*) = -11.4830;
 **Shock** tms(*"Olis"*,*"CHN"*,*"JPN"*) = -0.2359;
 **Shock** tms(*"Animals"*,*"CHN"*,*"JPN"*) = -6.7705;
 **Shock** tms(*"Milk"*,*"CHN"*,*"JPN"*) = -0.2146;
 **Shock** tms(*"Fishing"*,*"CHN"*,*"JPN"*) = -1.9260;
 **Shock** tms(*"BandT"*,*"CHN"*,*"JPN"*) = -2.9050;
 **Shock** tms(*"Others"*,*"CHN"*,*"JPN"*) = -4.8242;
 **Shock** tms(*"Cereals"*,*"CHN"*,*"KOR"*) = -7.2362;
 **Shock** tms(*"Fruit"*,*"CHN"*,*"KOR"*) = -50.3235;
 **Shock** tms(*"Suger"*,*"CHN"*,*"KOR"*) = -28.1428;
 **Shock** tms(*"Olis"*,*"CHN"*,*"KOR"*) = -64.2358;
 **Shock** tms(*"Animals"*,*"CHN"*,*"KOR"*) = -7.0170;
 **Shock** tms(*"Milk"*,*"CHN"*,*"KOR"*) = -26.8444;
 **Shock** tms(*"Fishing"*,*"CHN"*,*"KOR"*) = -11.9232;
 **Shock** tms(*"BandT"*,*"CHN"*,*"KOR"*) = -13.6182;
 **Shock** tms(*"Others"*,*"CHN"*,*"KOR"*) = -24.0873;
 **Shock** tms(*"Cereals"*,*"CHN"*,*"SEAsia"*) = -3.9542;
 **Shock** tms(*"Fruit"*,*"CHN"*,*"SEAsia"*) = -0.6305;
 **Shock** tms(*"Suger"*,*"CHN"*,*"SEAsia"*) = -0.4469;
 **Shock** tms(*"Animals"*,*"CHN"*,*"SEAsia"*) = -2.4538;
 **Shock** tms(*"Fishing"*,*"CHN"*,*"SEAsia"*) = -0.0016;
 **Shock** tms(*"BandT"*,*"CHN"*,*"SEAsia"*) = -13.3103;
 **Shock** tms(*"Others"*,*"CHN"*,*"SEAsia"*) = -2.0954;

S4

*! Begin CMFSTART file***servants**=1;
**start with** **MMNZ** = 40000000 ;

*! End CMFSTART file*
**auxiliary** **files** = .\CODE\GTAP;
**Solution** **file** = .\s-4 ;
**Method** = **euler**;
**Steps** = 50;

**accuracy** **percent** = 90;
**verbal** **description** = .\autosim rr-22_35;
**File** GTAPSETS = .\**DATA**\**sets**.har; *! constant input***File** GTAPDATA = dat-17_21.har; *! updated input***File** GTAPPARM = .\**DATA**\**default**.prm ; *! constant input***updated** **file** GTAPDATA = .\dat-s-3.har;
**intermediate** **extra** **data** =.\s-4 ;
**log file**=.\s-4.log;
**CPU**=**YES**;

*!neq= yes; !use if you want to check for errors in tab and cmf files!
!but dont wanna involve the data and calculations!
! Begin Common Closure file***Exogenous**
 pop
 psaveslack pfactwld
 profitslack incomeslack endwslack
 cgdslack tradslack
 ams atm atf ats atd
 aosec aoreg avasec avareg
 afcom afsec afreg afecom afesec afereg
 afall afeall aoall
 au dppriv dpgov dpsave
 **to** tp tm tms tx txs
 qo(ENDW_COMM,REG);

**Rest** **endogenous**;

*!swap aoreg(reg)=qgdp(reg);*
*! End Common Closure file*
*! The BASE Case MACRO Shocks
! --------------------
!*
*! Begin Period-Specific Shocks file
!==== Macro shocks ============================================================!***shock** POP(reg) = **select** **from** **file** POPCEPII_2.HAR **header** *"GROW"* slice *"Y2035"* ;
*!shock qgdp(reg) = select from file GDPCEPII.HAR header "GROW" slice "Y2027";***shock** qo(*"unsklab"*,reg) = **select** **from** **file** UNSKLSHKLI_2.HAR **header** *"YR35"* ;
**shock** qo(*"sklab"*,reg) = **select** **from** **file** SKLSHKLI_2.HAR **header** *"YR35"*;
**shock** qo(*"Capital"*,reg) = **select** **from** **file** CAPSHKLi_2.HAR **header** *"YR35"* ;
*!============================================================================!*
**shock** qo(*"natres"*,reg)=**uniform** 9.1; *!.7% per years, with exponentional growth
!1 0.7
!2 1.449
!3 2.25043
!4 3.1079601*
*! End Period-Specific Shocks file***Shock** tms(*"Cereals"*,*"NZL"*,*"CHN"*) = -0.2781;
 **Shock** tms(*"Fruit"*,*"NZL"*,*"CHN"*) = -2.6852;
 **Shock** tms(*"Suger"*,*"NZL"*,*"CHN"*) = -0.2062;
 **Shock** tms(*"Olis"*,*"NZL"*,*"CHN"*) = 0.0000;
 **Shock** tms(*"Animals"*,*"NZL"*,*"CHN"*) = -1.6781;
 **Shock** tms(*"Milk"*,*"NZL"*,*"CHN"*) = -1.6126;
 **Shock** tms(*"Fishing"*,*"NZL"*,*"CHN"*) = 0.0000;
 **Shock** tms(*"BandT"*,*"NZL"*,*"CHN"*) = 0.0000;
 **Shock** tms(*"Others"*,*"NZL"*,*"CHN"*) = -0.0001;
 **Shock** tms(*"Cereals"*,*"AUS"*,*"CHN"*) = -0.1870;
 **Shock** tms(*"Fruit"*,*"AUS"*,*"CHN"*) = -10.4354;
 **Shock** tms(*"Suger"*,*"AUS"*,*"CHN"*) = -10.3994;
 **Shock** tms(*"Olis"*,*"AUS"*,*"CHN"*) = -5.0821;
 **Shock** tms(*"Animals"*,*"AUS"*,*"CHN"*) = -3.2376;
 **Shock** tms(*"Milk"*,*"AUS"*,*"CHN"*) = -2.9920;
 **Shock** tms(*"Fishing"*,*"AUS"*,*"CHN"*) = -6.2012;
 **Shock** tms(*"BandT"*,*"AUS"*,*"CHN"*) = -10.2706;
 **Shock** tms(*"Others"*,*"AUS"*,*"CHN"*) = -5.1284;
 **Shock** tms(*"Cereals"*,*"SEAsia"*,*"CHN"*) = -0.4036;
 **Shock** tms(*"Fruit"*,*"SEAsia"*,*"CHN"*) = -0.0001;
 **Shock** tms(*"Suger"*,*"SEAsia"*,*"CHN"*) = -8.0158;
 **Shock** tms(*"Olis"*,*"SEAsia"*,*"CHN"*) = -1.3171;
 **Shock** tms(*"Animals"*,*"SEAsia"*,*"CHN"*) = 0.0000;
 **Shock** tms(*"Milk"*,*"SEAsia"*,*"CHN"*) = -0.2734;
 **Shock** tms(*"Fishing"*,*"SEAsia"*,*"CHN"*) = 0.0000;
 **Shock** tms(*"BandT"*,*"SEAsia"*,*"CHN"*) = -4.4746;
 **Shock** tms(*"Others"*,*"SEAsia"*,*"CHN"*) = -0.6597;
 **Shock** tms(*"Cereals"*,*"JPN"*,*"CHN"*) = -0.4827;
 **Shock** tms(*"Fruit"*,*"JPN"*,*"CHN"*) = -1.7084;
 **Shock** tms(*"Suger"*,*"JPN"*,*"CHN"*) = 0.0000;
 **Shock** tms(*"Olis"*,*"JPN"*,*"CHN"*) = -9.6693;
 **Shock** tms(*"Animals"*,*"JPN"*,*"CHN"*) = -3.7812;
 **Shock** tms(*"Milk"*,*"JPN"*,*"CHN"*) = -13.7500;
 **Shock** tms(*"Fishing"*,*"JPN"*,*"CHN"*) = -3.3648;
 **Shock** tms(*"BandT"*,*"JPN"*,*"CHN"*) = -7.1773;
 **Shock** tms(*"Others"*,*"JPN"*,*"CHN"*) = -7.1347;
 **Shock** tms(*"Cereals"*,*"KOR"*,*"CHN"*) = -0.5741;
 **Shock** tms(*"Fruit"*,*"KOR"*,*"CHN"*) = -11.9383;
 **Shock** tms(*"Suger"*,*"KOR"*,*"CHN"*) = 0.0000;
 **Shock** tms(*"Olis"*,*"KOR"*,*"CHN"*) = -9.9984;
 **Shock** tms(*"Animals"*,*"KOR"*,*"CHN"*) = -8.0651;
 **Shock** tms(*"Milk"*,*"KOR"*,*"CHN"*) = -3.0610;
 **Shock** tms(*"Fishing"*,*"KOR"*,*"CHN"*) = -5.1362;
 **Shock** tms(*"BandT"*,*"KOR"*,*"CHN"*) = -5.3622;
 **Shock** tms(*"Others"*,*"KOR"*,*"CHN"*) = -6.7406;
 **Shock** tms(*"Cereals"*,*"CHN"*,*"AUS"*) = 0.0000;
 **Shock** tms(*"Fruit"*,*"CHN"*,*"AUS"*) = -0.1375;
 **Shock** tms(*"Suger"*,*"CHN"*,*"AUS"*) = -2.9521;
 **Shock** tms(*"Olis"*,*"CHN"*,*"AUS"*) = -0.2780;
 **Shock** tms(*"Animals"*,*"CHN"*,*"AUS"*) = -1.2781;
 **Shock** tms(*"Fishing"*,*"CHN"*,*"AUS"*) = 0.0000;
 **Shock** tms(*"BandT"*,*"CHN"*,*"AUS"*) = -1.0122;
 **Shock** tms(*"Others"*,*"CHN"*,*"AUS"*) = -1.5847;
 **Shock** tms(*"Cereals"*,*"CHN"*,*"JPN"*) = -2.0446;
 **Shock** tms(*"Fruit"*,*"CHN"*,*"JPN"*) = -3.2556;
 **Shock** tms(*"Suger"*,*"CHN"*,*"JPN"*) = -11.4830;
 **Shock** tms(*"Olis"*,*"CHN"*,*"JPN"*) = -0.2359;
 **Shock** tms(*"Animals"*,*"CHN"*,*"JPN"*) = -6.7705;
 **Shock** tms(*"Milk"*,*"CHN"*,*"JPN"*) = -0.2146;
 **Shock** tms(*"Fishing"*,*"CHN"*,*"JPN"*) = -1.9260;
 **Shock** tms(*"BandT"*,*"CHN"*,*"JPN"*) = -2.9050;
 **Shock** tms(*"Others"*,*"CHN"*,*"JPN"*) = -4.8242;
 **Shock** tms(*"Cereals"*,*"CHN"*,*"KOR"*) = -7.2362;
 **Shock** tms(*"Fruit"*,*"CHN"*,*"KOR"*) = -50.3235;
 **Shock** tms(*"Suger"*,*"CHN"*,*"KOR"*) = -28.1428;
 **Shock** tms(*"Olis"*,*"CHN"*,*"KOR"*) = -64.2358;
 **Shock** tms(*"Animals"*,*"CHN"*,*"KOR"*) = -7.0170;
 **Shock** tms(*"Milk"*,*"CHN"*,*"KOR"*) = -26.8444;
 **Shock** tms(*"Fishing"*,*"CHN"*,*"KOR"*) = -11.9232;
 **Shock** tms(*"BandT"*,*"CHN"*,*"KOR"*) = -13.6182;
 **Shock** tms(*"Others"*,*"CHN"*,*"KOR"*) = -24.0873;
 **Shock** tms(*"Cereals"*,*"CHN"*,*"SEAsia"*) = -3.9542;
 **Shock** tms(*"Fruit"*,*"CHN"*,*"SEAsia"*) = -0.6305;
 **Shock** tms(*"Suger"*,*"CHN"*,*"SEAsia"*) = -0.4469;
 **Shock** tms(*"Animals"*,*"CHN"*,*"SEAsia"*) = -2.4538;
 **Shock** tms(*"Fishing"*,*"CHN"*,*"SEAsia"*) = -0.0016;
 **Shock** tms(*"BandT"*,*"CHN"*,*"SEAsia"*) = -13.3103;
 **Shock** tms(*"Others"*,*"CHN"*,*"SEAsia"*) = -2.0954;
 **Shock** ams(TRAD_COMM,REG,*"CHN"*) = **uniform** 5;

Other code:

exogenous

pop

psaveslack pfactwld

profitslack incomeslack endwslack

cgdslack tradslack

ams atm atf ats atd

aosec aoreg avasec avareg

afcom afsec afreg afecom afesec afereg

aoall afall afeall

au dppriv dpgov dpsave

to tp tm tms tx txs

qo(ENDW_COMM,REG) ;

Rest Endogenous ;

Swap afereg( REG) = qgdp( REG)

Swap afreg( REG) = qcgds( REG)
