# Supplementary material for: Impacts of RCEP’s trade barrier reductions on China’s agricultural trade: A GTAP simulation
Source: PLoS One. 2025 Jul 10;20(7):e0328060. doi: 10.1371/journal.pone.0328060 (PMC12244828; doi:10.1371/journal.pone.0328060)
Supplement: S3 File — (DOCX) [file pone.0328060.s003.docx]

**Dynamic GTAP Model: Unveiling the Temporal Transmission of Policies and the Dynamic Evolution of the Economy**

The dynamic GTAP (Global Trade Analysis Project) model incorporates capital accumulation, labor market adjustments, and the cumulative effects of policy shocks into a multi-period equilibrium framework by introducing a time-recursive structure. The capital stock is updated annually using the perpetual inventory method, creating an intertemporal linkage between investment and depreciation. The growth of skilled and unskilled labor is projected based on educational attainment and demographic extrapolation, respectively. Policy shocks, such as tariff reductions and China’s accession to the WTO, are implemented gradually over different time periods, and their effects are cumulatively integrated into the long-term economic trajectory through recursive mechanisms.

The theoretical distinction between the dynamic GTAP model and the standard closed-form conditions lies in the treatment of the time dimension and the introduction of intertemporal dynamics. The standard GTAP model relies on comparative static analysis, focusing on economic equilibrium at a single point in time. It simulates the immediate effects of policy changes by exogenously setting the savings rate, capital stock, and labor structure. For example, tariff reductions in a static framework directly adjust trade flows and prices, but their impact is confined to the current economic structure, failing to capture long-term feedback mechanisms such as capital accumulation, technology diffusion, or human capital enhancement. Although this simplification facilitates computation, it sacrifices the accurate portrayal of the dynamic evolution of the economy.

In contrast, the dynamic GTAP model employs a recursive dynamic framework that endogenizes time as a core dimension of the model, constructing a multi-period interlinked economic system. Its theoretical foundation lies in the intertemporal allocation and accumulation mechanisms: the capital stock is updated annually using the perpetual inventory method, reflecting the continuous interaction between investment and depreciation. The labor market differentiates between skilled and unskilled workers, with the former based on educational attainment projections and the latter dynamically adjusted according to demographic trends. Policy shocks are decomposed into progressively implemented sub-processes, with the effects of each stage cumulatively integrated through recursive mechanisms. For example, the tariff reductions associated with China’s accession to the WTO not only stimulate exports in the short term but also reshape the global value chain in the long term by attracting foreign investment, increasing the capital stock, and generating technology spillovers. This design enables the dynamic model to reveal how policies transmit through time, triggering industrial structural transformation, productivity growth, and changes in trade patterns, thus more closely approximating the gradual and path-dependent nature of real-world economic dynamics. Its theoretical advantage lies in integrating the intertemporal decision-making of economic agents with the temporal path of policies, providing a microeconomic foundation and macroeconomic coherence for analyzing long-term challenges such as climate change and population transitions.
